# Heart failure potentially affects the cortical structure of the brain

**DOI:** 10.18632/aging.205762

**Published:** 2024-04-22

**Authors:** Yinqin Hu, Tianyun Shi, Zhaohui Xu, Meng Zhang, Jiahui Yang, Zhirui Liu, Qiqi Wan, Yongming Liu

**Affiliations:** 1 Department of Cardiovascular Disease, Shuguang Hospital Affiliated to Shanghai University of Traditional Chinese Medicine, Shanghai, China; 2 Anhui Provincial Hospital of Integrated Medicine, Anhui Hospital of Shuguang Hospital Affiliated to Shanghai University of TCM, Hefei 230011, Anhui, China

**Keywords:** psycho-cardiology, heart failure, cerebral cortex structure, Mendelian randomization, causal effect

## Abstract

Background: Heart failure (HF) has been reported to affect cerebral cortex structure, but the underlying cause has not been determined. This study used Mendelian randomization (MR) to reveal the causal relationship between HF and structural changes in the cerebral cortex.

Methods: HF was defined as the exposure variable, and cerebral cortex structure was defined as the outcome variable. Inverse-variance weighted (IVW), MR-Egger regression and weighted median (WME) were performed for MR analysis; MR-PRESSO and Egger’s intercept was used to test horizontal pleiotropy; and “leave-one-out” was used for sensitivity analysis.

Results: Fifty-two single nucleotide polymorphisms (SNPs) were defined as instrumental variables (IVs), and there was no horizontal pleiotropy in the IVs. According to the IVW analysis, the OR and 95% CI of cerebral cortex thickness were 0.9932 (0.9868-1.00) (P=0.0402), and the MR-Egger intercept was -15.6× 10^-5^ (P = 0.7974) and the Global test pval was 0.078. The P-value of the cerebral cortex surface was 0.2205, and the MR-Egger intercept was -34.69052 (P= 0.6984) and the Global Test pval was 0.045. HF had a causal effect on the surface area of the caudal middle frontal lobule (P=0.009), insula lobule (P=0.01), precuneus lobule (P=0.049) and superior parietal lobule (P=0.044).

Conclusions: HF was potentially associated with changes in cortical thickness and in the surface area of the caudal middle frontal lobule, insula lobule, precuneus lobule and superior parietal lobule.

## INTRODUCTION

Heart failure (HF) is the severe or terminal stage of various heart diseases and is characterized by high morbidity, mortality, and hospitalization rates; poor quality of life; and high medical costs [1]. The data show that the global incidence of HF ranges from 1% to 3%. Due to population aging, the treatment and prognosis of ischemic heart disease have improved, and effective evidence-based treatment has prolonged the survival of patients with HF. However, the incidence of HF has continued to increase, ranging from 1/1000 person-years to 20/1000 person-years, and the prevalence in different regional populations also differs. The incidence of HF in European and American populations ranges from 2/1000 person-years to 3/1000 person-years, and the incidence of HF is positively correlated with age [2]. Heart failure can lead to reduced pumping function of the heart muscle and reduced blood flow throughout the body, resulting in insufficient blood supply to the brain. In fact, abnormal cerebral hemodynamics may lead to a lack of glucose and oxygen in the brain, which in turn leads to a series of adverse biochemical events, ultimately leading to metabolic and tissue damage, which is a major cause of structural changes in the brain [3]. The structural changes in the cerebral cortex caused by this phenomenon are also related mainly to cognitive dysfunction, such as vascular dementia and Alzheimer’s disease. Studies have shown that patients with coronary heart disease exhibit a wide range of gray matter density decreases, while patients with heart failure mainly exhibit a significant decrease in gray matter density in the posterior, middle and precuneus regions of the cingulate gyrus [4]. Moreover, the cortical thickness of the frontal, parietal, temporal and occipital lobes is reduced in patients with heart failure, which mainly controls autonomic, cognitive, emotional, language and visual functions. With the atrophy of these functional cortical areas, patients also exhibit corresponding neurological dysfunction [5]. Brain structure is closely related to heart failure, and the existing research is limited mainly to the study of brain structure changes caused by hemodynamics; however, there is no clear targeted research on whether there is a genetic link between heart failure and cerebral cortex structure.

Randomized controlled trials (RCTs) are not implemented clinically due to various limiting factors, and observational experimental methods can lead to biased study results due to confounding factors and reverse causation, resulting in relatively low credibility. Mendelian randomization (MR) involves an analysis of genetic variables that follow Mendel’s law of inheritance and exploits single nucleotide polymorphisms (SNPs) as instrumental variables (IVs) to infer the causality of an observed association between a modifiable exposure and a clinically relevant outcome [6]. Alleles are randomly separated during meiosis, so MR can reduce the bias caused by confounding factors [7]. In addition, since genetic variation occurs before disease and the order of the two cannot be reversed, MR can also avoid the interference of reverse causality [7].

This study was based on a large sample genome-wide association study (GWAS; GE-Nanowide Association Study). SNP sites published in the GWAS database were used as instrumental variables of genetic variation to explore the causal relationship between HF and cerebral cortex structure through a two-sample MR research method.

## MATERIALS AND METHODS

### Study design

**Figure 1:**
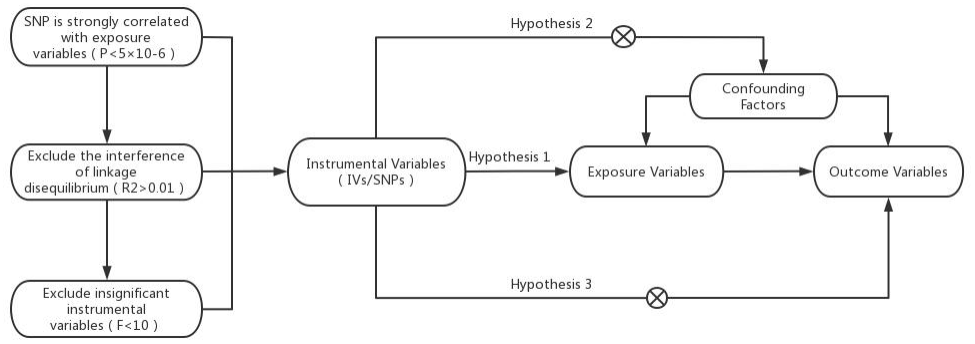
Model of the Mendelian randomization analysis.

In this study, heart failure was used as an exposure factor, and single nucleotide polymorphisms (SNPs) that were significantly correlated with heart failure were used as instrumental variables (IVs). The cerebral cortex structure was selected as the outcome. The TwoSampleMR package in R was used to conduct causal association analysis, and the Cochran Q heterogeneity test, pleiotropy test and sensitivity analysis were performed to verify the reliability of the results. MR analysis relies on three assumptions, as shown in Figure 1 [8]: (1) IVs are strongly correlated with exposure variables, (2) IVs are not associated with confounding factors affecting exposure outcomes, and (3) IVs affect outcome factors only through exposure factors.

### Data sources

We searched for GWAS data on HFs and the cerebral cortex on the website ‘https://gwas.mrcieu.ac.uk/datasets’. The data for HF (ebi-a-GCST009541) were derived from the GWAS statistical results published in 2020 and included a sample of 977,323 people, 47,309 cases and 930,014 controls, and 7,773,021 SNPs. In this study, GWAS data related to cortical structure were obtained from the ENIGMA study, a genome-wide association meta-analysis of brain magnetic resonance imaging data from 51,665 people; the surface area, average thickness, and 34 known functional regions of the entire cortex were analyzed. The HF patients with cerebral cortex structure data included in this study were mostly European.

### Instrumental variables

We obtained HF-related genetic data from the website ‘https://gwas.mrcieu.ac.uk/datasets’ using R software. To avoid analytical bias caused by linkage disequilibrium (LD) between SNPs, we used all SNPs that were significant (*P* < 5×10^-8^) and predicted significant genome-wide exposure. SNPs with an R^2^ value >0.001 and a base physical distance less than 10,000 kb were removed, and SNPs with the smallest P-values were retained. Candidate SNPs were matched with GWAS data for the outcome variable based on the chromosome and location. To further evaluate weak IV bias, the F statistic was used to calculate the power of the IV. When the IVs with an F value <10 were eliminated, the specific calculation formula was F=β^2^/SE^2^, where β^2^ is the effect value of the β allele and SE is the standard error. Since only 12 significant and independent SNPs with genome-wide significance remained after screening, we used a higher P-value (P < 5×10^-6^) to obtain SNPs predicting HF as the final IV included in the study.

### MR and sensitivity analysis

The analysis methods used in this study were mainly the inverse-variance weighted (IVW) method in the TwoSampleMR package [9, 10], MR-Egger regression [11] and the weighted median (WME) method [12].

The heterogeneity test tests the difference between various IVs. If *P* was >0.05, there was no heterogeneity. In this study, the *P-*value of the Cochran Q test was used to assess heterogeneity. A *P*-value <0.05 indicated heterogeneity. In contrast, *P*>0.05 indicated no heterogeneity. The pleiotropy test [13] verifies the reliability of MR analysis results and is often performed via the MR-PRESSO test and the intercept term of the MR-Egger regression method. *P*>0.05 was considered to indicate no horizontal pleiotropy; if pleiotropy was indicated, the MR analysis results were not reliable. The “leave-one-out” method [14] was adopted to test the sensitivity. The principle is to gradually eliminate the results of a single SNP to determine whether the results are outliers and to observe the stability and reliability of the results after the removal of each SNP.

### Availability of data and materials

All data are publicly available.

### Consent for publication

Written informed consent for publication was obtained from all participants.

## RESULTS

### Instrumental variables

In this study, HF was taken as an exposure factor, R software was used to screen SNPs with genome-wide significance according to the screening criteria, and a total of 52 SNPs were included as IVs. Table 1 shows the 15 most significant SNPs, and the remaining complete SNP data are shown in Supplementary Table 1.

**Table 1:** Basic information of the SNPs associated with HF.

**SNPs**	**CHR**	**POS**	**Other allele**	**Effect allele**	***β***	***SE***	***P***
rs55751848	1	57018257	C	G	-0.0425	0.0089	1.79E-06
rs593467	1	70584460	G	A	-0.0548	0.0118	3.42E-06
rs660240	1	109817838	T	C	0.0611	0.0097	3.00E-10
rs35054810	1	222722282	G	A	0.0725	0.0143	3.98E-07
rs7559452	2	3885011	A	G	0.0468	0.0102	4.47E-06
rs17496249	2	37102249	A	G	-0.0372	0.0079	2.49E-06
rs12477245	2	107584422	C	T	0.1192	0.0236	4.40E-07
rs7369998	2	125815568	G	A	-0.059	0.0126	2.83E-06
rs72844714	2	133386122	C	A	0.0559	0.0121	3.84E-06
rs80087882	2	201379864	G	A	0.0609	0.0125	1.10E-06
rs4376020	3	5397743	T	A	-0.0612	0.0123	6.50E-07
rs9815816	3	85930582	T	C	0.0479	0.0099	1.31E-06
rs10938398	4	45186139	G	A	0.0389	0.008	1.16E-06
rs11722972	4	69897984	T	G	-0.0519	0.0114	5.30E-06
rs2634071	4	111669220	T	C	-0.0923	0.0101	6.33E-20

SNPs, single nucleotide polymorphisms; CHR, chromosome; POS, location; EA, effector allele; OA, noneffector allele. β, standard error. SE, standard error of beta.

### MR analysis of HF and cortical thickness

**Figure 2:**
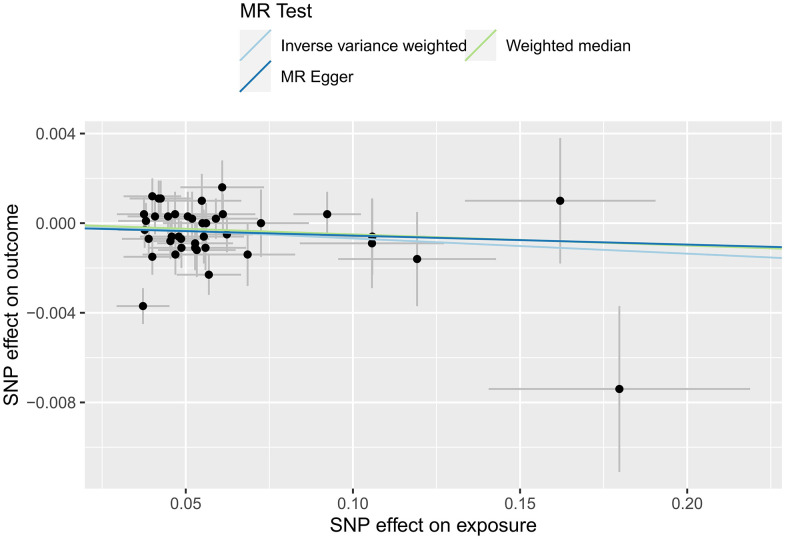
MR scatter plot of cortical thickness in HF patients.

In this study, IVW regression, MR-Egger regression and WME analysis in the TwoSampleMR package were used to perform MR analysis of HF and cerebral cortex thickness. Table 2 shows the results of the MR analysis, and the scatter plot is shown in Figure 2. IVW analysis revealed *β*=-0.0068, *SE*=0.0033, *P*=0.0402 and an *OR (95% CI*)=0.9932 (0.9868-1.00); MR-Egger analysis revealed *β*=-0.004, *SE*=0.0114, *P*=0.7273, and an *OR (95% CI*)=0.996 (0.974-1.0185). WME analysis revealed *β*=-0.005, *SE*=0.0041, *P*=0.2223, and *OR (95% CI*)=0.995 (0.9871-1.003). Although the results of the MR-Egger and WME analyses showed that HF had no significant effect on the thickness of the cerebral cortex, the results of the IVW analysis showed *P*=0.0402. In addition, the *β* values of the IVW, MR-Egger and WME analyses were in the same direction, and the IVW results prevailed. Moreover, we used the MR-Egger intercept to verify the presence of pleiotropy in this study. The results showed an Egger’s intercept value of -15.6× 10-5, which is infinitely close to 0, *SE* = 6.04× 10-4, *P* = 0.7974 and the global test pval=0.078, indicating that horizontal pleiotropy did not exist. There was no multi-effect interference in the MR results. Therefore, these results suggest that HF has a significant effect on the thickness of the cerebral cortex, that there is a causal relationship between the two, and that the thickness of the cerebral cortex is negatively correlated with the incidence of HF.

**Table 2:** MR results of cortical thickness in HF patients.

**MR method**	***β***	**SE**	***OR (95% CI)***	***P***
IVW	-0.0068	0.0033	0.9932 (0.9868-1.00)	0.0402
MR–Egger	-0.004	0.0114	0.9960 (0.974-1.0185)	0.7273
Weighted median	-0.005	0.0041	0.995 (0.9871-1.003)	0.2223

MR, Mendelian randomization; β, beta; SE, standard error; OR, odds ratio; IVW, inverse-variance weighted.

### MR analysis of the HF and cortical surface

**Figure 3:**
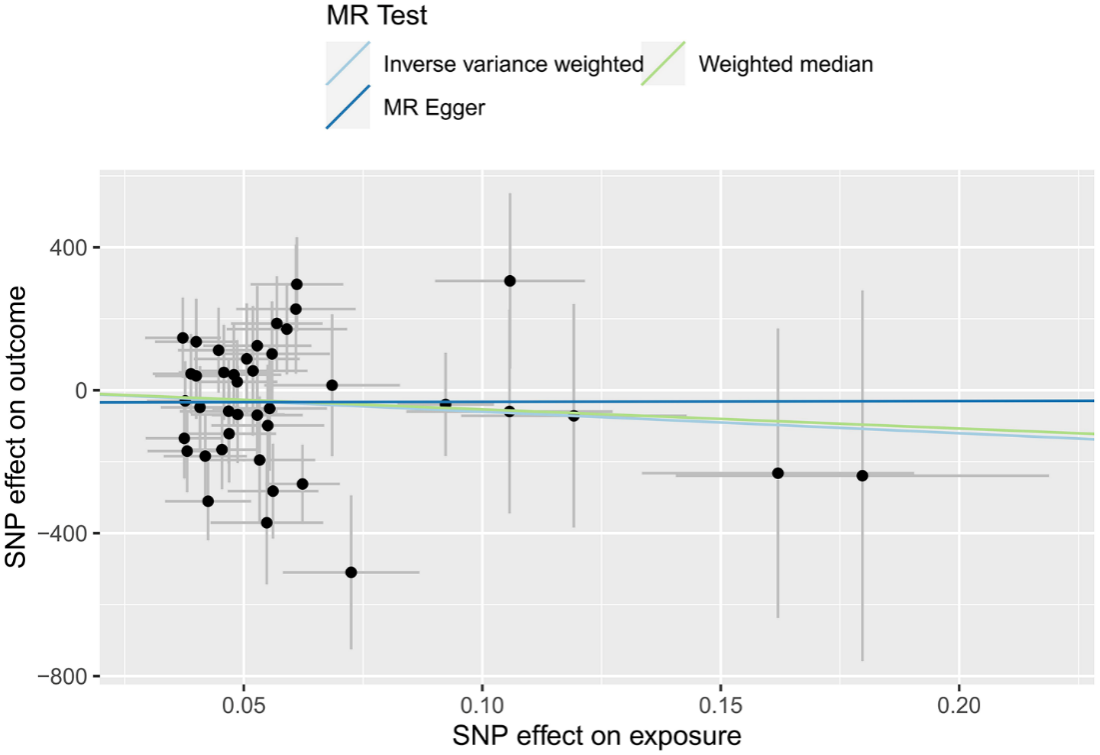
MR scatter plot of the effect of HF on the cerebral cortex surface area.

In this study, IVW, MR-Egger regression and WME analyses in the TwoSampleMR package were used to perform MR analysis of HFs and the surface area of the cerebral cortex. Table 3 shows the results of the MR analysis, and the scatter plot is shown in Figure 3. IVW analysis revealed *β*=-601.7768, *SE*=491.1655, *P*=0.2205, and *OR (95% CI*)=4.483756E-262 (0-5.497039E +156); MR-Egger analysis revealed *β*=23.0309, *SE*=1675.8267, *P*=0.9891, and *OR (95% CI*)=1.005105e+10 (0-inf); and WME analysis showed *β*=-535.222, *SE*=641.9665, *P*=0.4044, and *OR (95% CI*)=3.597802E-233 (0-inf). According to the results of the IVW, MR-Egger and WME analyses, none of the three analysis methods showed statistical significance, suggesting that there was no direct causal relationship between the cerebral cortex surface area and HF incidence. Moreover, we used the MR-Egger intercept to verify the presence of pleiotropy in this study, and the results showed Egger’s intercept=-34.69052, *SE* =88.86852, *P* = 0.6984, and a global test pval=0.045, indicating that heart failure and cerebral cortex surface area may be pleiotropic.

**Table 3:** MR results of the effect of HF on cortical surface area.

**MR method**	***β***	**SE**	***OR (95% CI)***	***P***
IVW	-601.7768	491.1655	4.483756E-262 (0-5.497039e+156)	0.2205
MR–Egger	23.0309	1675.8267	1.005105e+10 (0-inf)	0.9891
Weighted median	-535.222	641.9665	3.597802E-233 (0-inf)	0.4044

MR, Mendelian randomization; *β*, beta; *SE*, standard error; *OR*, odds ratio; IVW, inverse-variance weighted.

### MR analysis of the HF and cerebral cortex

**Figure 4:**
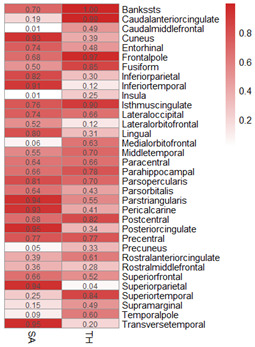
MR heatmap of the effect of HF on the structure of the cerebral cortex.

In this study, according to 34 specific cerebral cortical functional areas with known functions defined by the Desikan-Killiany atlas [15], the IVW analysis method in the TwoSampleMR package was used to conduct MR analysis on the effects of HF on the structure of cerebral cortical functional areas. In this section, we present the MR analysis results for 34 functional brain regions using the global weighted method because the global weighted method may be less affected by neuroanatomical variation between different individuals [16]. Figure 4 and Supplementary Table 2 show the results of this part of the study, suggesting that HF had a significant impact on the surface area of the caudal middle frontal lobule (*P*=0.009), insula lobule (*P*=0.01), precuneus lobule (*P*=0.049) and thickness of the superior parietal lobule (*P*=0.044). This difference was statistically significant. A scatter diagram of the MR analysis of the structure of various functional areas of the cerebral cortex in HF patients is shown (Supplementary Figures 1–3, 8–10). The pleiotropic analysis of the effects of HF on the structure of various functional areas of the brain is shown in Supplementary Tables 3, 4.

### Sensitivity analysis of the HF and cerebral cortex structure

**Figure 5:**
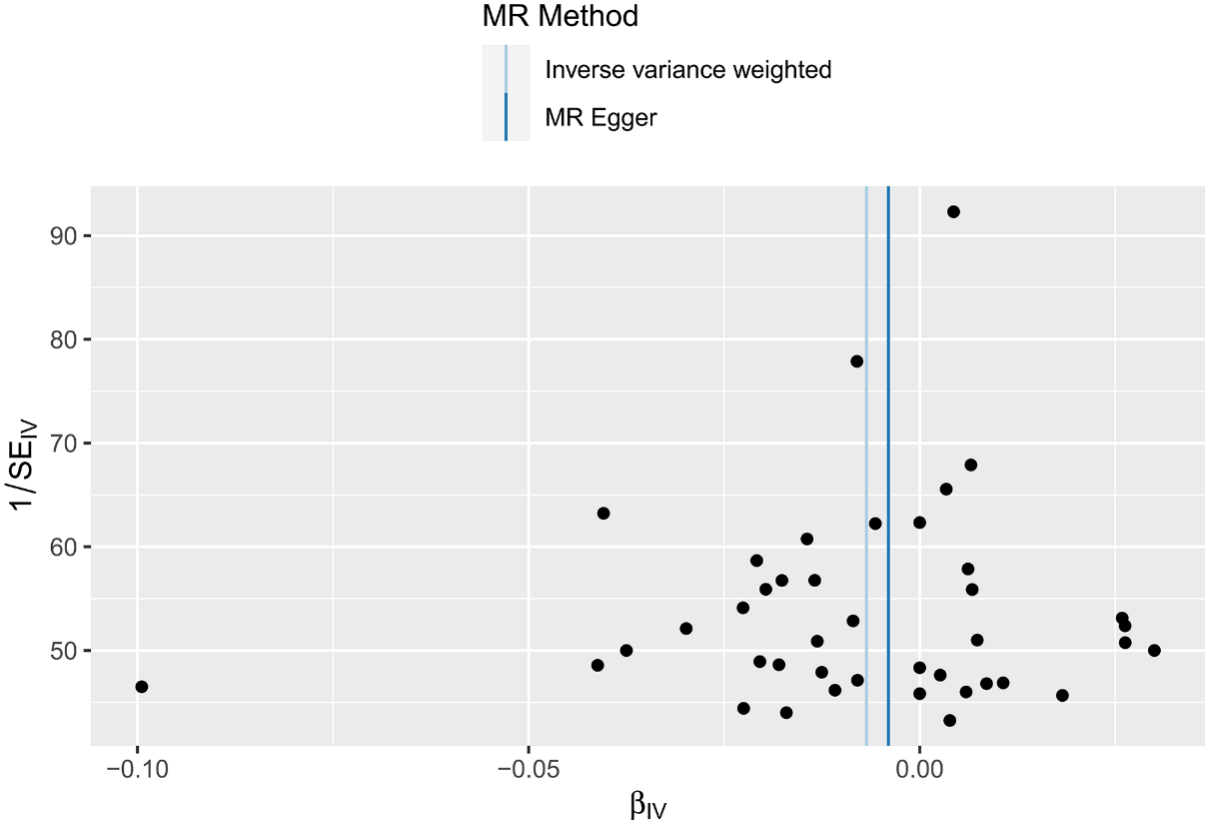
Funnel plot of the heterogeneity test results for the effect of HF on cerebral cortex thickness according to the MR method.

This study strictly followed the screening criteria for IVs, and most of the included participants were European; therefore, the possibility of false negative results was unlikely, and the results were tested for heterogeneity. The IVW test of the effect of HF on cerebral cortex thickness yielded Cochran’s Q=53.8265 and *P*=0.071; the MR-Egger test yielded Cochran’s Q=53.7344 and *P*=0.058. All *P*-values were >0.05, indicating no heterogeneity. The results are shown in Figure 5.

**Figure 6:**
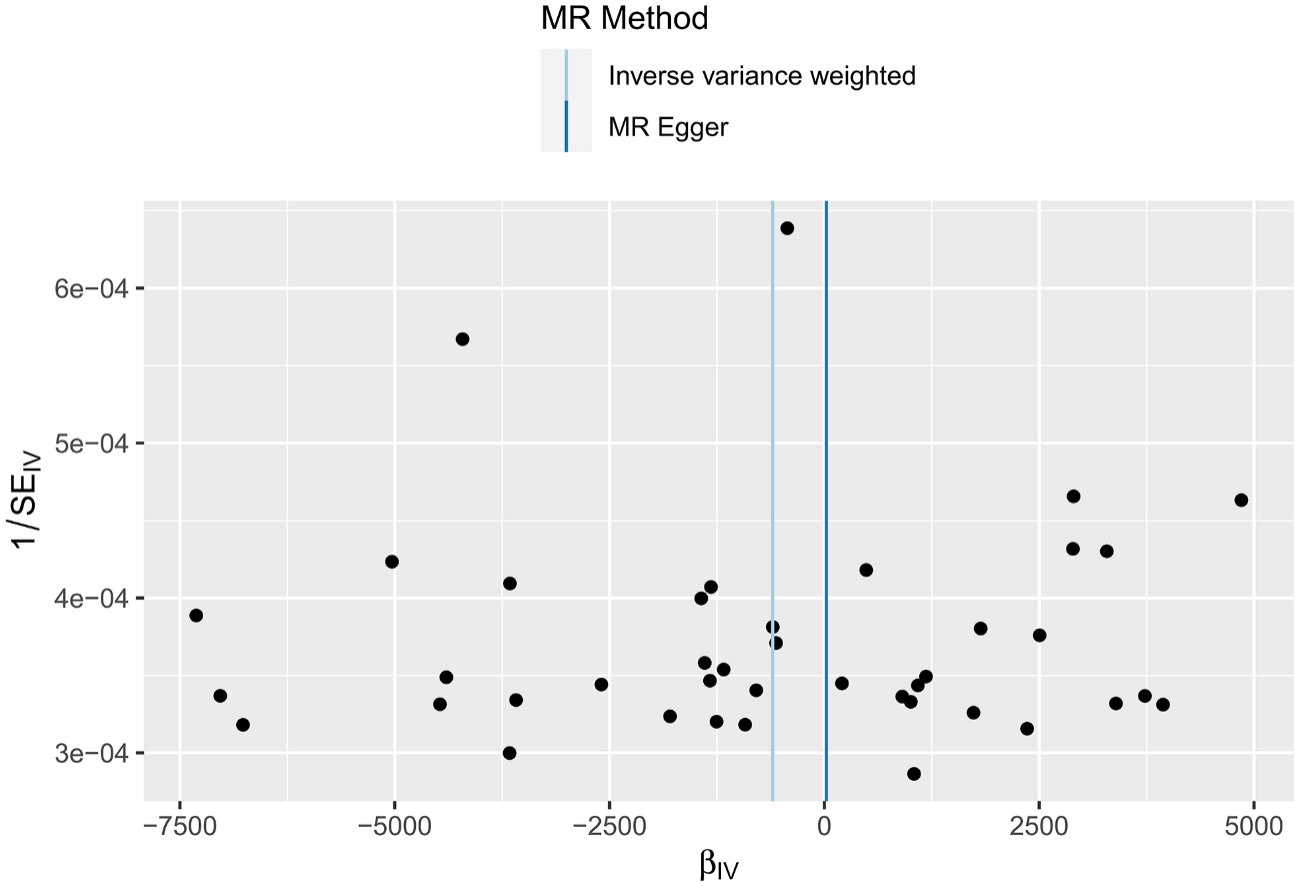
Funnel plot of the heterogeneity test results of the MR analysis of the relationship between HF and the cerebral cortex surface area.

The IVW test of the effect of HF on the cerebral cortex surface area yielded Cochran’s Q=56.6142 and *P*=0.042; the MR-Egger test yielded Cochran’s Q=56.3939 and *P*=0.035. All P-values were <0.05, indicating heterogeneity. The results are shown in Figure 6. A funnel plot for the analysis of cortical heterogeneity in the relationship between various functional areas of the brain and HF is shown in Supplementary Figures 4, 5, 11, 12 and Supplementary Tables 5, 6.

**Figure 7:**
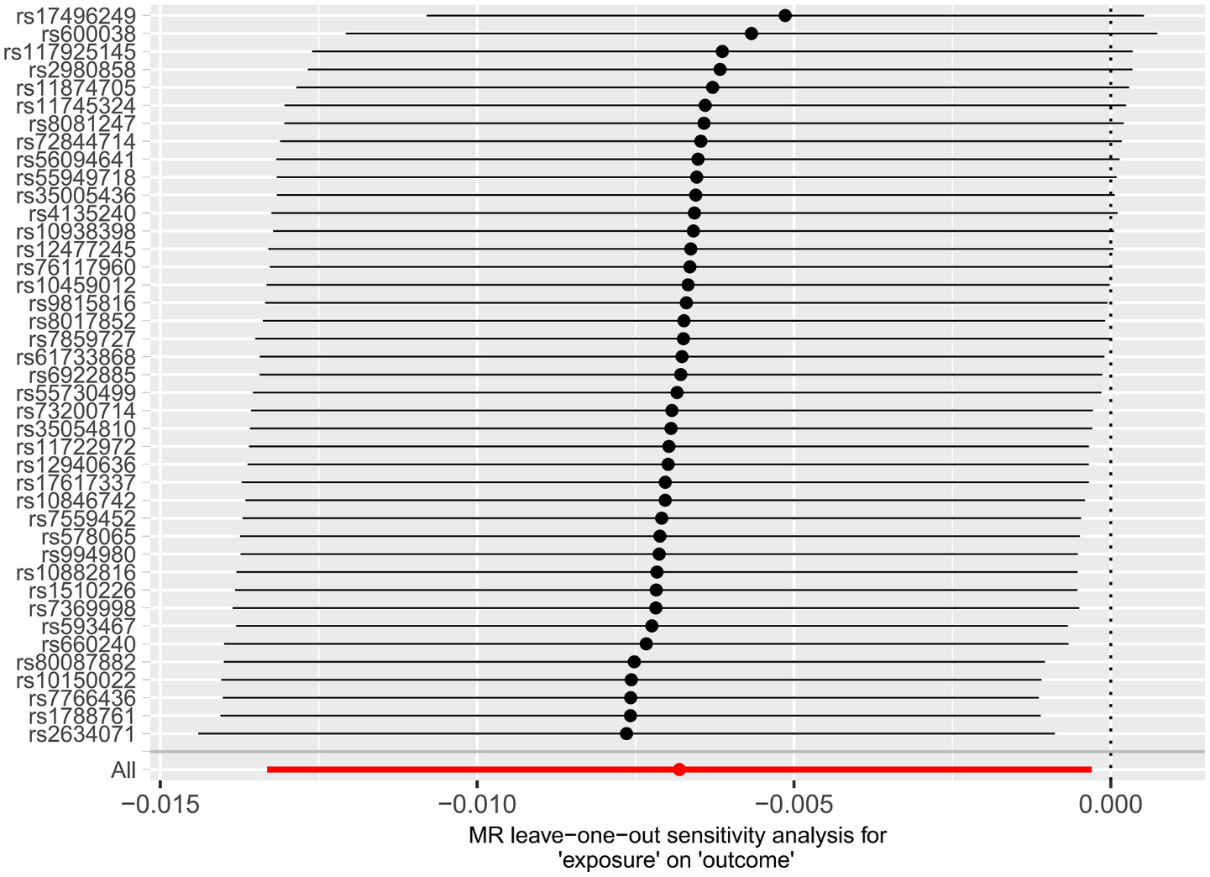
Forest plot of the “leave-one-out” method in the MR analysis of the relationship between HF and cerebral cortex thickness.

**Figure 8:**
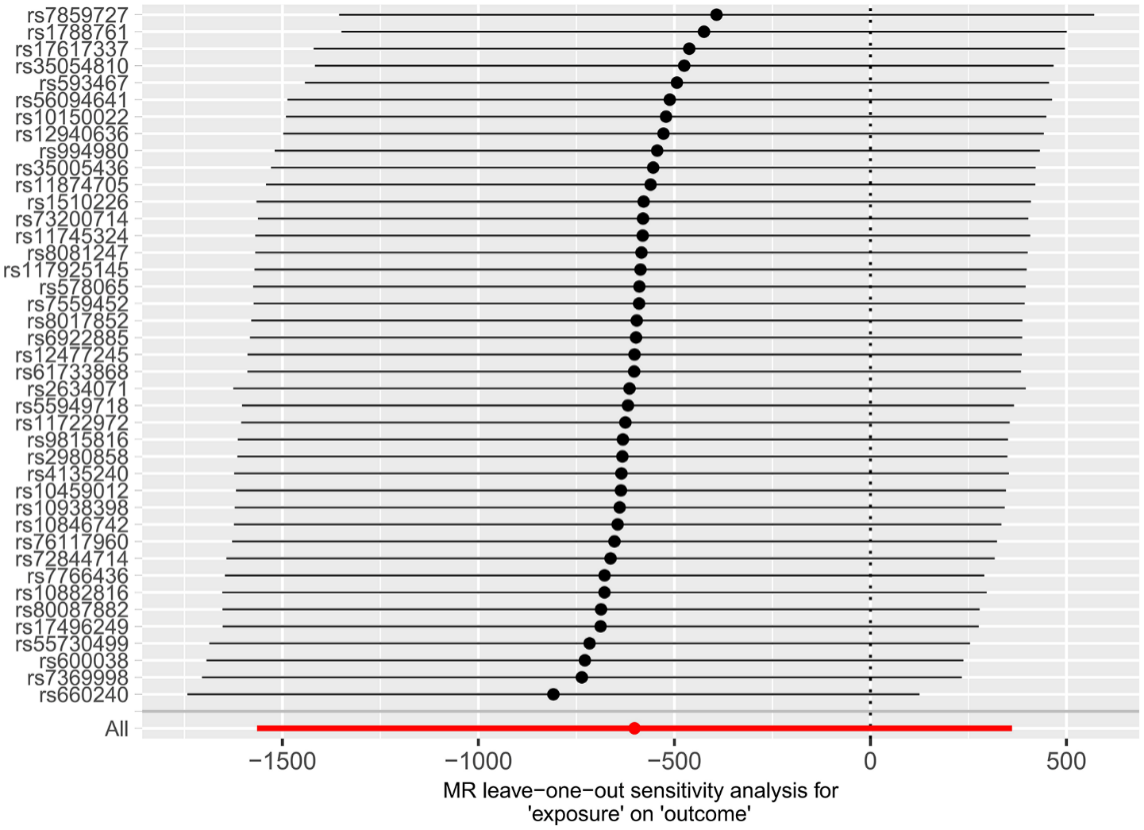
Forest plot of the “leave-one-out” method used in the MR analysis of the relationship between HF and cerebral cortex surface area.

Subsequently, we applied the “leave-one-out” method of sensitivity analysis to visualize the IVW analysis results of the relationship between HF and cerebral cortex structure (Figures 7, 8). After the above significant and independent SNPs were sequentially excluded, the IVW outcome effect values of the remaining SNPs did not significantly fluctuate. All the SNPs were close to the red dot position in the forest plot, and all the *P-*values were >0.05, indicating that there was no SNP in the IVs that strongly influenced the results; this finding showed that the results obtained by the IVW analysis method were stable and reliable. The sensitivity analysis results of the effects of HF on the various functional areas of the brain are shown in Supplementary Figures 6, 7, 13, 14.

## DISCUSSION

At present, numerous observational studies have shown that cerebral cortex structure is related to HF incidence, but there is no evidence showing whether there is a causal relationship between the two conditions. This study systematically identified a causal relationship between HF and cerebral cortex structure using two-sample MR analysis. Our findings suggest that HF affects cortical thickness, specifically the surface area of the caudal middle frontal lobule, insula, precuneus lobule, and superior parietal lobule. Moreover, sensitivity analysis did not reveal pleiotropy, further confirming the stability of the conclusion. This difference may be related to the reduced pumping function of the heart muscle in patients with HF.

Cortical structure, specifically cortical thickness, is considered a neuroimaging biomarker for predicting cognitive decline. Moreover, it is also believed that the surface area of the cerebral cortex is the key to reducing cortical volume in patients with cognitive or mental disorders [17]. Moreover, numerous studies have suggested that cortical surface area may be more sensitive than cortical thickness for the prediction of mental illness [18–20]. The middle frontal gyrus, where the caudal middle frontal lobule is located, is a region of the ventral prefrontal cortex that has been recognized as an important brain region leading to depression and is responsible for many cognitive functions. Examples of emotion processing include decision processing, emotional cognition [21], working memory [22], attention processing [23, 24], and top-down regulation in emotional processing [25]. Moreover, the prefrontal regions (BA8, BA9, BA10, BA46, and BA47), where the medial frontal gyrus is located, are located in front of the motor and premotor areas. These functional areas are related to human personality and determine a person’s social and moral consciousness and emotional depth [26].

The insula, located deep in the lateral sulci of the brain, is also known as the “Island of Reil” and is located deep in the temporal lobe. The insula was originally described as the paralimbic or limbic integration cortex [27]. Analysis of nearly 1,800 functional neuroimaging experiments revealed that the insula is divided into four main functional regions: the sensorimotor, central-olfactogustatory, socioemotional and cognitive anterior-dorsal regions [28]. The anterior insula, as the cortical center for visceral information processing and perception, is believed to play a crucial role in emotional experiences and subjective feelings [29]. Neural function imaging has shown that the expression of negative emotions is also related to the activation of the insula [30]. Moreover, patients with insular lobe injury exhibit various changes in subjective emotions, mainly manifested as anxiety-related indifference [31, 32]. The insula may play an important role in the management of social emotions.

The precuneus is a functional region of the parietal lobe; as one of the core regions of the default network, the precuneus is involved in the human body’s situational memory, self-focused attention, visuospatial intention and self-emotional processing [33]. We found that the functional connectivity of the Broadman region 47/12 of the lateral orbitofrontal cortex was enhanced with the precuneus, angular gyrus and Broadman region 21 of the visual cortex. This enhanced nonreward or punishment system (Brodmann: BA47 and areas 12) with functional connections to the precuneus and angular gyrus is associated with explicit emotional negative self-awareness and self-esteem in individuals with depression [34]. The posterior cingulate cortex and anterior cuneus are considered hubs of the default mode network (DMN) and are involved in social cognition and theory of mind. Moreover, a previous study revealed that the functional connectivity of the posterior cingulate gyrus, anterior cuneus and angular gyrus is related to the severity of depression [35]. In addition, studies have shown that the superior lobular cortex plays an important role in cognitive control and detail attention [36, 37].

Subsequently, we analyzed the SNPs significantly associated with heart failure identified in the present study and observed that the expression of GPR39 corresponding to rs72844714 decreased in the hippocampus and cortex of patients with depression [38]. Zinc, a stimulator of GPR39, can activate the Gαs pathway, leading to an increase in cyclic adenosine monophosphate (cAMP) and activation of protein kinase A (PKA). This pathway can result in phosphorylation of cAMP response element-binding protein (CREB) and an increase in cAMP response element (CRE)-dependent transcription. Consequently, BDNF leads to the upregulation of brain-derived neurotrophic factor (BDNF) and tropomyosin receptor kinase B (TrkB) in neurons [39]. A zinc-deficient diet for 6 weeks can reduce the protein expression of GPR39 and BDNF in the prefrontal cortex [40]. After prolonged antidepressant treatment, GPR39 is upregulated, where it exerts antidepressant effects through the Gαq pathway via the CREB/BDNF/TrkB pathway [41–43]. Simultaneously, zinc can activate postsynaptic GPR39-mediated increases in intracellular calcium [44]. Calcium release, through upregulation of the postsynaptic membrane KCC2 (K+/Cl−) cotransporter protein, increases K-dependent Cl− efflux in postsynaptic cells, thereby enhancing inhibitory tone and preventing excitotoxicity [44–46].

Notably, GPR39 is closely associated with the dopaminergic and 5-HT systems. When mice are treated with tyrosine hydroxylase inhibitor (alphaMT) and tryptophan hydroxylase inhibitor (pCPA) to block dopamine and 5-HT synthesis, GPR39 in the prefrontal cortex is significantly upregulated [47, 48]. Furthermore, the striatin (STRN) gene corresponding to rs17496249 is widely expressed in the striatum and serves as a regulator of striatal neuron development; moreover, this gene is significantly relevant for diagnosing symptomatic depression in patients with subsyndromal syndromes and severe depressive disorders [50, 51]. The FTO gene corresponding to rs56094641 is highly enriched in the cortex and hippocampus. Interaction with CaMKII delays the dephosphorylation of CREB in human neuroblastoma cells [52]. CaMKII-mediated activation of CREB promotes the transcription and translation of the key neuronal plasticity proteins SYN and PSD95 [53, 54], potentially influencing the occurrence of depression. Further research has confirmed the neuroprotective role of hippocampal FTO in depression-like behavior through the activation of the CaMKII/CREB signaling pathway, improving hippocampal synaptic plasticity (dendritic remodeling, PSD95, and SYN expression) [55].

The structural changes in the cerebral cortex caused by HF may lead to a range of neuropsychiatric symptoms in patients. The link between psychosocial factors and CVD incidence has been identified as an important public health problem that mainly includes psychiatric symptoms such as anxiety and depression and can increase the occurrence of major adverse cardiovascular events [56]. Depression and anxiety have been shown to be prevalent in approximately 15%-20% of CVD patients and can coexist for a long time [57]. This, together with the results of this study, also confirms the theory of “psycho-cardiology”. The concept of “psycho-cardiology” began with an article published in the American Journal of Psychosomatics in 1985 titled “Psychocardiology: meeting place of the heart and mind” [58]. Therefore, we suggest that timely and appropriate mental health education and treatment should be given to patients with HF and other heart diseases to reduce the incidence of various psychiatric symptoms and improve the prognosis of patients with CVD.

**Figure 9:**
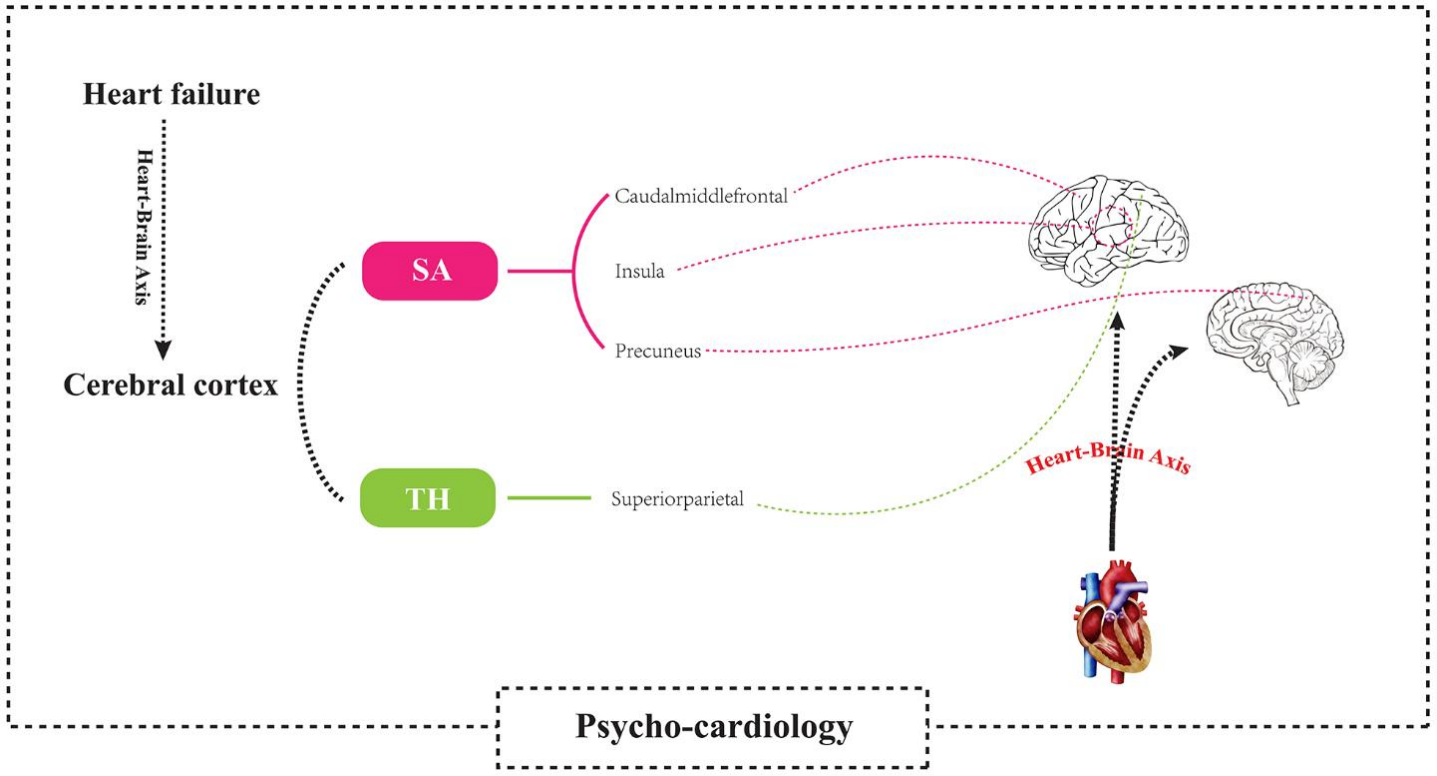
**Using a two-sample Mendelian randomization framework, we revealed that heart failure causally influences brain cortical structure alterations, supporting the existence of the “heart-brain axis”.** SA, Surficial area; TH, thickness.

This study explored the effect of HF on cerebral cortex structure at the genetic level. According to our review results, this study is the first to perform MR analysis on the causal relationship between HF and cerebral cortex structure. Our results showed that although there was a direct effect of HF only on the cortical thickness of the whole-brain structure, there was a direct causal relationship between HF and the surface areas of the caudal middle frontal lobule, insula lobule, and precuneus lobule and the cortical thickness of the superior parietal lobule. This result supports the causal relationship between cardiac injury and neurological dysfunction, providing systematic and strong evidence for the theory of the “heart-brain axis” and “psycho-cardiology” Figure 9.

This study has several limitations. First, our study was limited to individuals of European descent, so whether our findings are generalizable to other individuals of different ethnicities remains unknown. Second, in this two-sample MR study design, we were unable to determine whether there was sample overlap between the exposure and outcome factor populations of the included GWAS dataset, which could lead to bias in the results. Third, we did not distinguish the phenotypes of patients with HF in more detail, such as those with reserved ejection fraction, those with reduced ejection fraction, or those with intermediate ejection fraction; therefore, we did not explore the effects of these different phenotypes on cerebral cortex structure. Finally, although a series of methods were used to rule out potential confounders and outliers and the sensitivity analyses did not detect any pleiotropy, we still cannot completely rule out all potential pleiotropy. Given these limitations, additional research should be performed to better confirm these possible associations, especially the clinical outcomes reflected in these results.

## CONCLUSIONS

In summary, this study revealed a direct association between HF and cerebral cortex structure through comprehensive and systematic MR analysis. Our results showed that the surface area of the caudal middle frontal lobule, insula lobule, and precuneus lobule and the cortical thickness of the superior parietal lobule were directly affected by HF. Head MRI may be used for the early diagnosis and prediction of neuropsychiatric diseases in patients with HF. To some extent, this study provides a theoretical basis for theories of the “heart-brain axis” and “psycho-cardiology”. However, due to the limitations of this study, the specific mechanism of the “heart-brain axis” should be further investigated.

## Supplementary Material

Supplementary Figures

Supplementary Tables
