# Supplementary material for: Heart failure potentially affects the cortical structure of the brain
Source: Aging (Albany NY). 2024 Apr 22;16(8):7357–86. doi: 10.18632/aging.205762 (PMC11087114; doi:10.18632/aging.205762)
Supplement: Supplementary Figures [file aging-16-205762-s001.pdf]

## SUPPLEMENTARY FIGURES

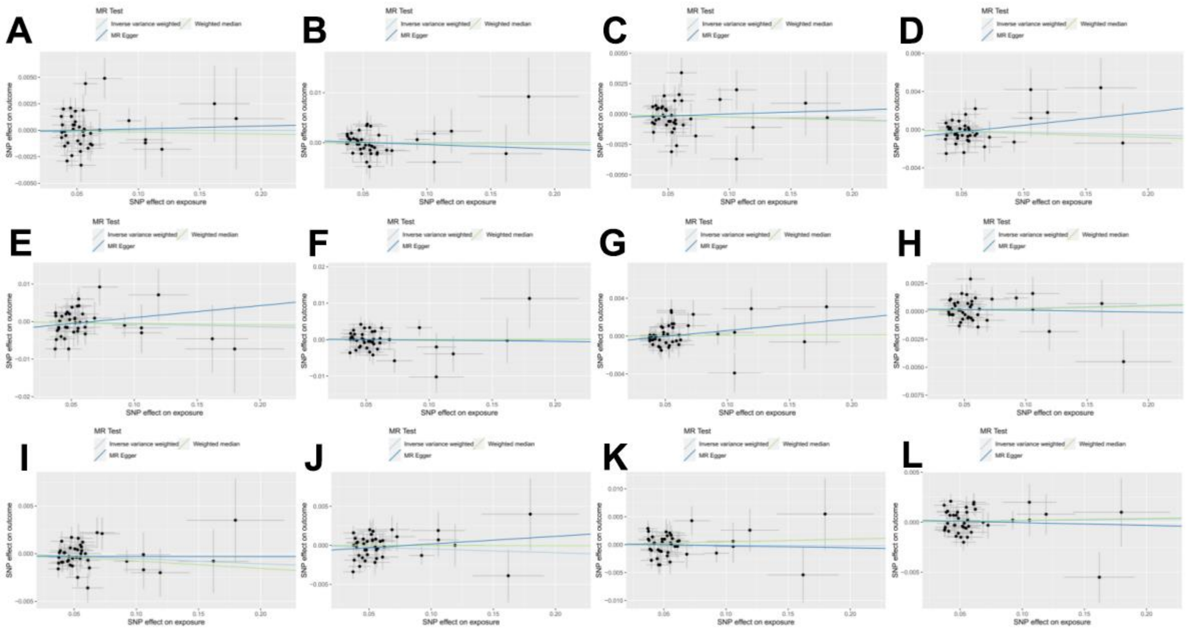


**Supplementary Figure 1. MR scatter plots of the relationship between heart failure and cortical thickness in cerebral functional areas 1.** (**A**) Banksst, (**B**) caudal anterior cingulate, (**C**) caudal middle frontal, (**D**) cuneus, (**E**) entorhinal, (**F**) frontal pole, (**G**) fusiform, (**H**) inferior parietal, (**I**) inferior temporal, (**J**) insula, (**K**) isthmus cingulate, (**L**) lateral occipital.

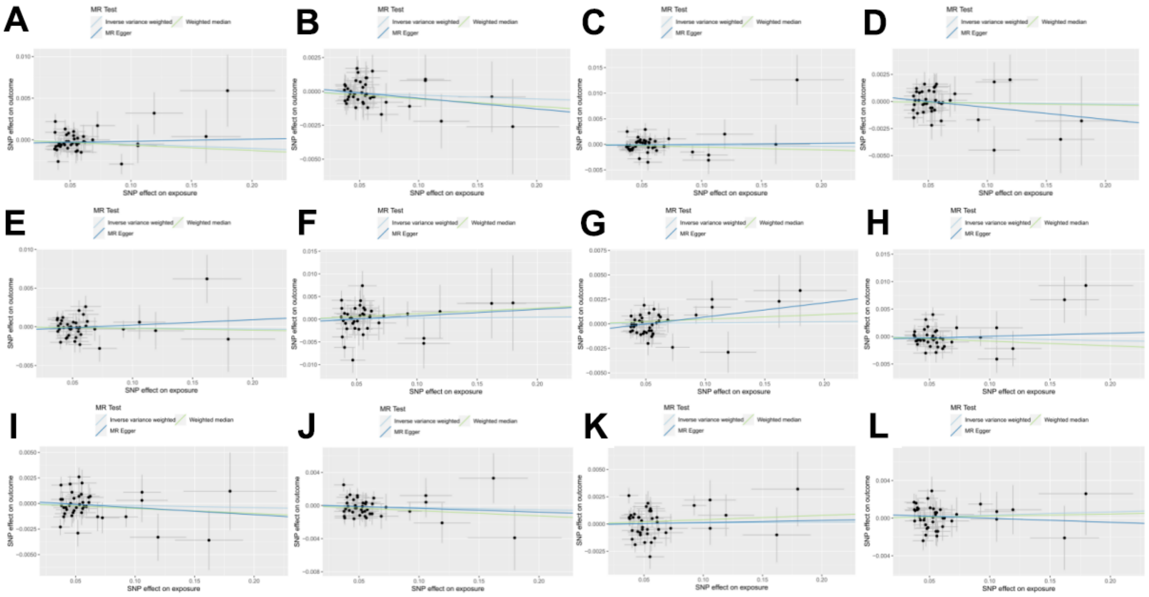


**Supplementary Figure 2. MR scatter plots of the relationship between heart failure and cortical thickness in cerebral functional areas 2.** (**A**) Lateral orbitofrontal, (**B**) lingual, (**C**) posterior cingulate medial orbitofrontal, (**D**) middle temporal, (**E**) paracentral, (**F**) parahippocampal, (**G**) pars opercularis, (**H**) pars orbitalis, (**I**) pars triangularis, (**J**) pericalcarine, (**K**) postcentral, (**L**) posterior cingulate.

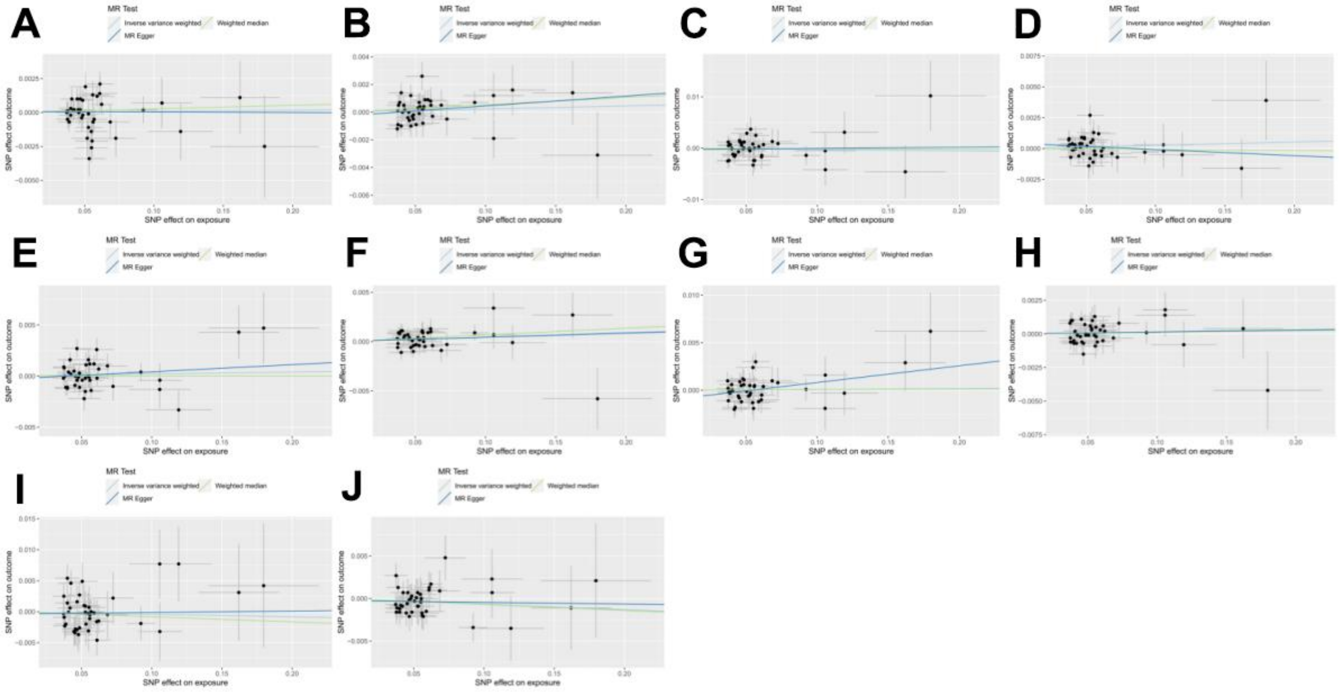


**Supplementary Figure 3. MR scatter plots of the relationship between heart failure and cortical thickness in cerebral functional areas 3.** (**A**) Precentral, (**B**) precuneus, (**C**) rostral anterior cingulate, (**D**) rostral middle frontal, (**E**) superior frontal, (**F**) superior parietal, (**G**) superior temporal, (**H**) supramarginal, (**I**) temporal pole, (**J**) transverse temporal.

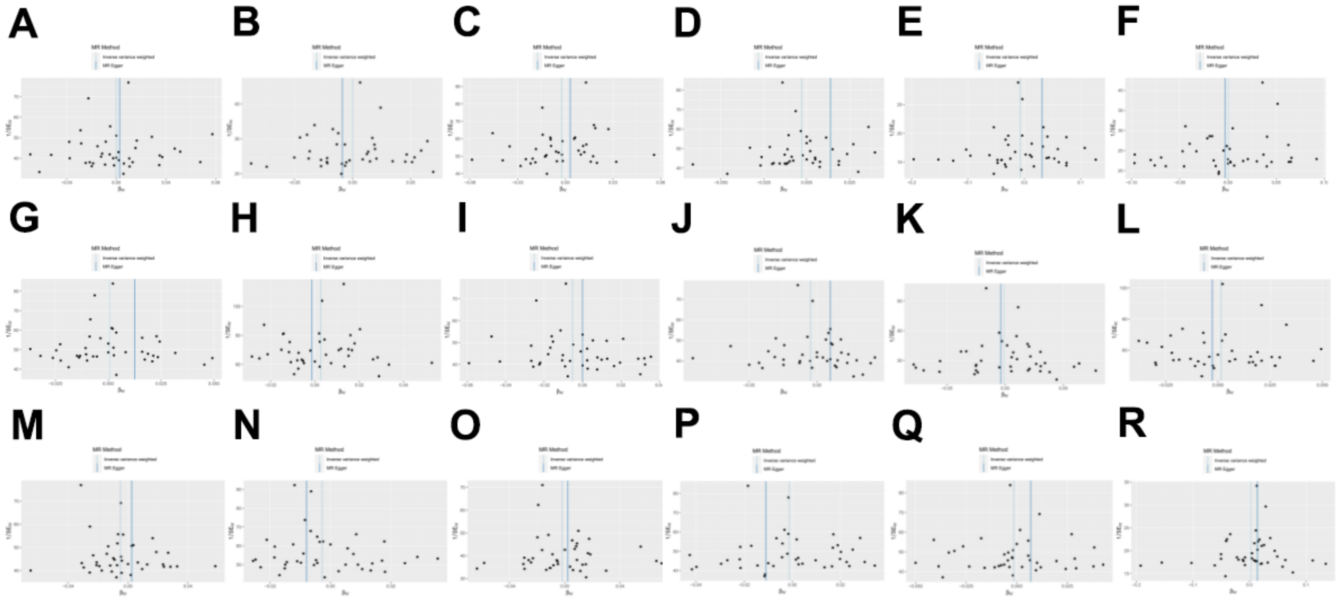


**Supplementary Figure 4. Funnel plot 1 of heterogeneity test results of the relationship between heart failure and cerebral functional area cortex thickness by MR.** (**A**) Bankssts, (**B**) caudal anterior cingulate, (**C**) caudal middle frontal, (**D**) cuneus, (**E**) entorhinal, (**F**) frontal pole, (**G**) fusiform, (**H**) inferior parietal, (**I**) inferior temporal, (**J**) insula, (**K**) isthmus cingulate, (**L**) lateral occipital, (**M**) lateral orbitofrontal, (**N**) lingual, (**O**) medial orbitofrontal, (**P**) middle temporal, (**Q**) paracentral, (**R**) parahippocampal.

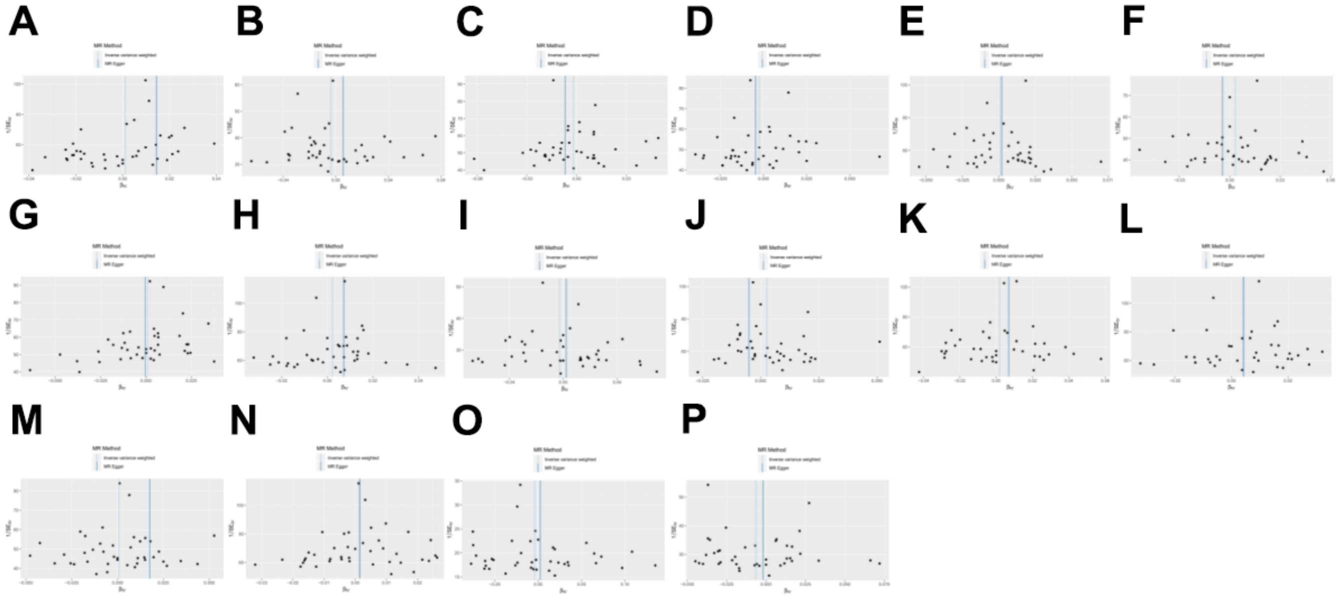


**Supplementary Figure 5. Funnel plot 2 of heterogeneity test results of the relationship between heart failure and cerebral functional area cortex thickness by MR.** (**A**) Pars opercularis, (**B**) pars orbitalis, (**C**) pars triangularis, (**D**) pericalcarine, (**E**) postcentral, (**F**) posterior cingulate, (**G**) precentral, (**H**) precuneus, (**I**) rostral anterior cingulate, (**J**) rostral middle frontal, (**K**) superior frontal, (**L**) superior parietal, (**M**) superior temporal, (**N**) supramarginal, (**O**) temporal pole, (**P**) transverse temporal.

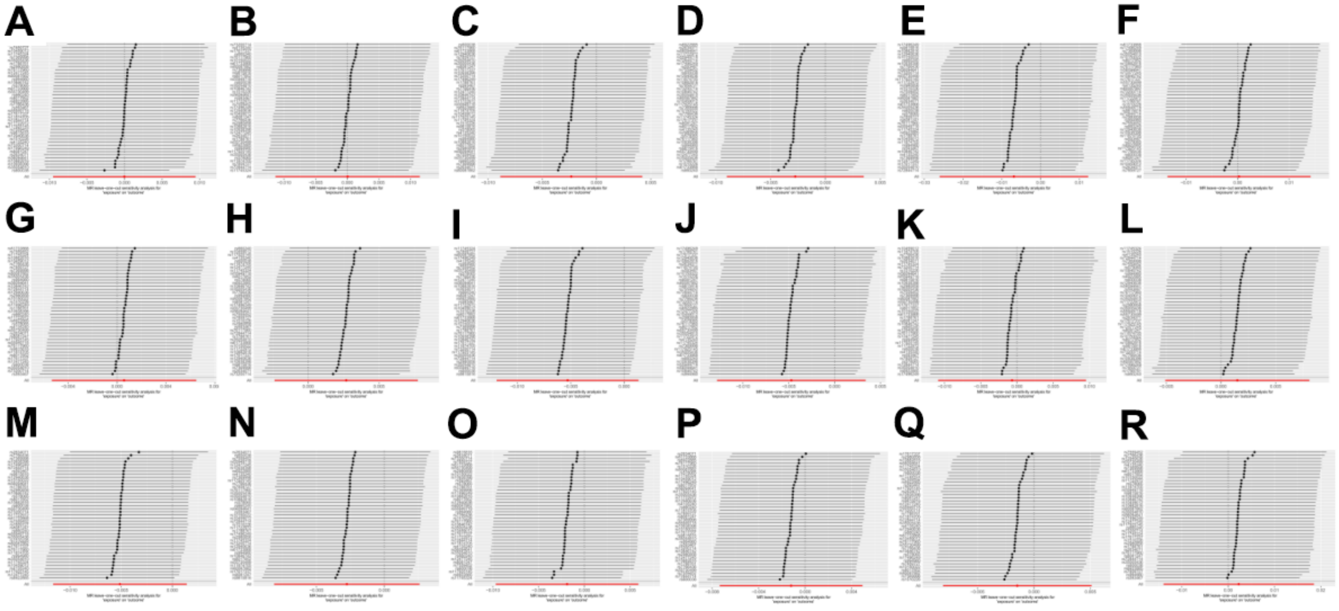


**Supplementary Figure 6. "Leave-one-out" forest map 1 of MR analysis of in the relationship between heart failure and the cortical thickness of brain functional areas.** (**A**) Bankssts, (**B**) caudal anterior cingulate, (**C**) caudal middle frontal, (**D**) cuneus, (**E**) entorhinal, (**F**) frontal pole, (**G**) fusiform, (**H**) inferior parietal, (**I**) inferior temporal, (**J**) insula, (**K**) isthmus cingulate, (**L**) lateral occipital, (**M**) lateral orbitofrontal, (**N**) lingual, (**O**) medial orbitofrontal, (**P**) middle temporal, (**Q**) paracentral, (**R**) parahippocampal.

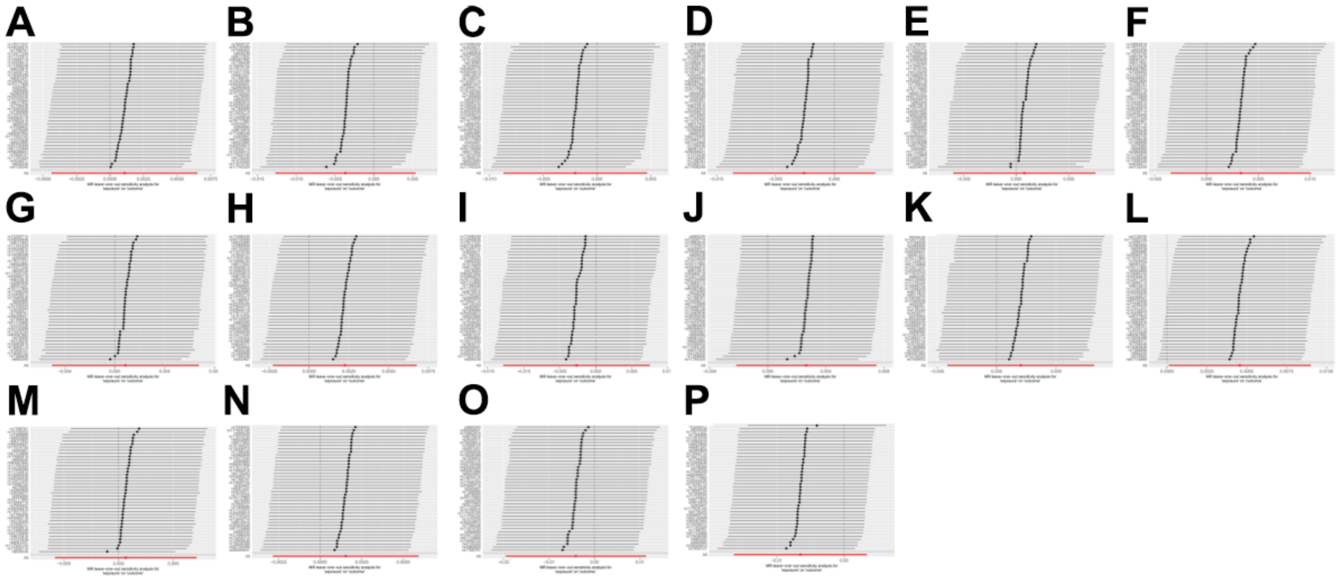


**Supplementary Figure 7. "Leave-one-out" forest map 2 of MR analysis of in the relationship between heart failure and the cortical thickness of functional brain areas.** (**A**) Pars opercularis, (**B**) pars orbitalis, (**C**) pars triangularis, (**D**) pericalcarine, (**E**) postcentral, (**F**) posterior cingulate, (**G**) precentral, (**H**) precuneus, (**I**) rostral anterior cingulate, (**J**) rostral middle frontal, (**K**) superior frontal, (**L**) superior parietal, (**M**) superior temporal, (**N**) supramarginal, (**O**) temporal pole, (**P**) transverse temporal.

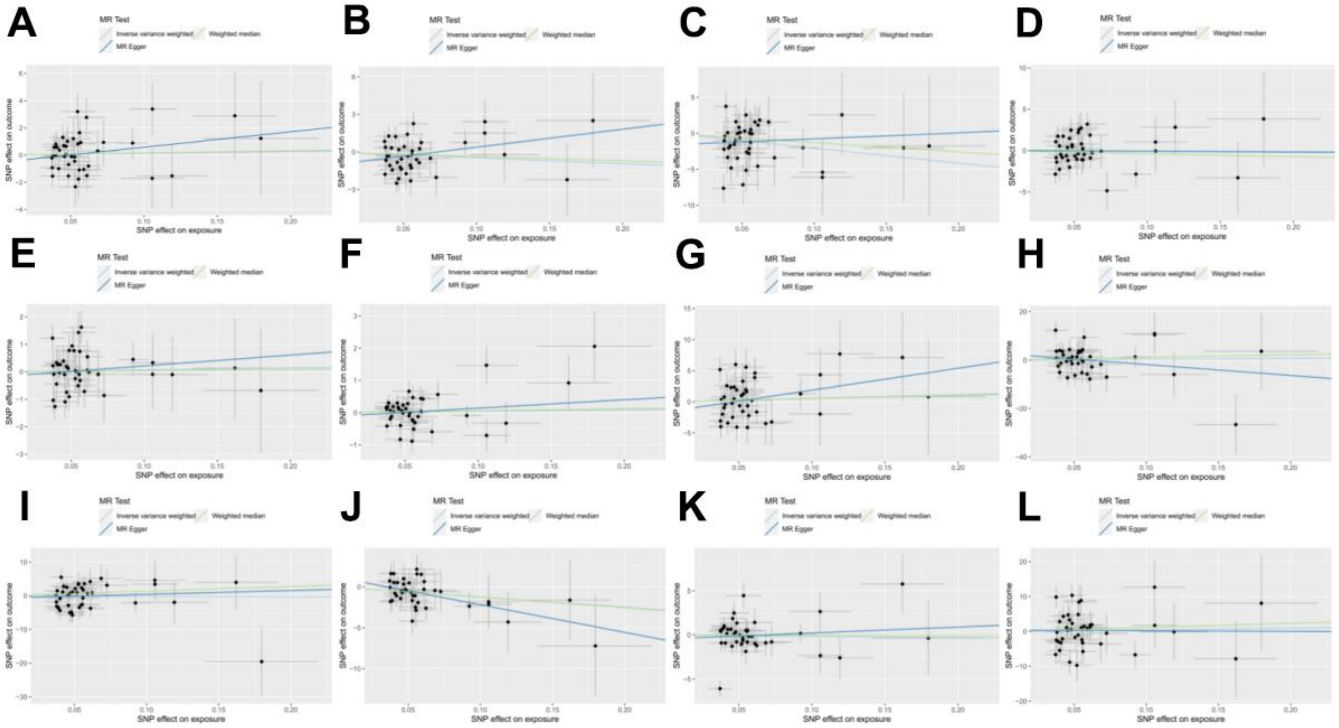


**Supplementary Figure 8. MR scatter plot 1 of the relationship between heart failure and the cortical surface area of functional brain areas.** (**A**) Banksst, (**B**) caudal anterior cingulate, (**C**) caudal middle frontal, (**D**) cuneus, (**E**) entorhinal, (**F**) frontal pole, (**G**) fusiform, (**H**) inferior parietal, (**I**) inferior temporal, (**J**) insula, (**K**) isthmus cingulate, (**L**) lateral occipital.

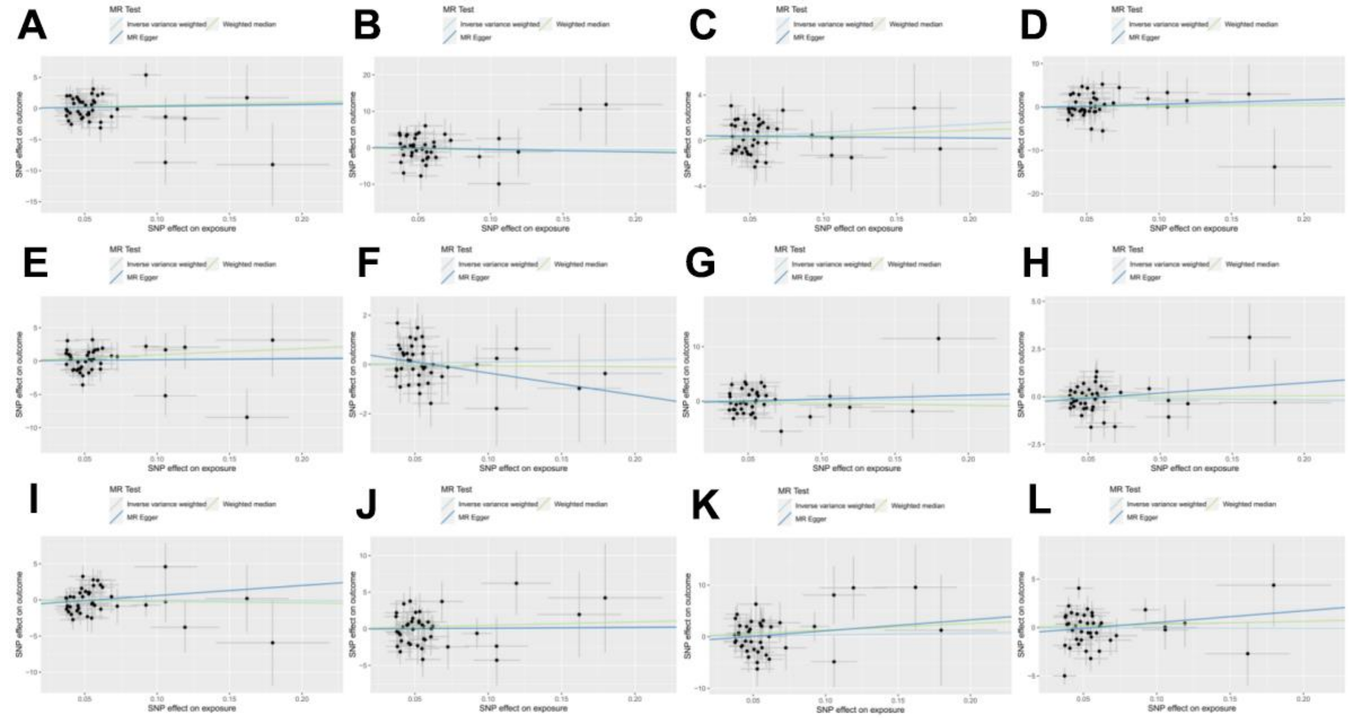


**Supplementary Figure 9. MR scatter plot 2 of the relationship between heart failure and the cortical surface area of functional brain areas.** (**A**) Lateral orbitofrontal, (**B**) lingual, (**C**) posterior cingulate medial orbitofrontal, (**D**) middle temporal, (**E**) paracentral, (**F**) parahippocampal, (**G**) pars opercularis, (**H**) pars orbitalis, (**I**) pars triangularis, (**J**) pericalcarine, (**K**) postcentral, (**L**) posterior cingulate.

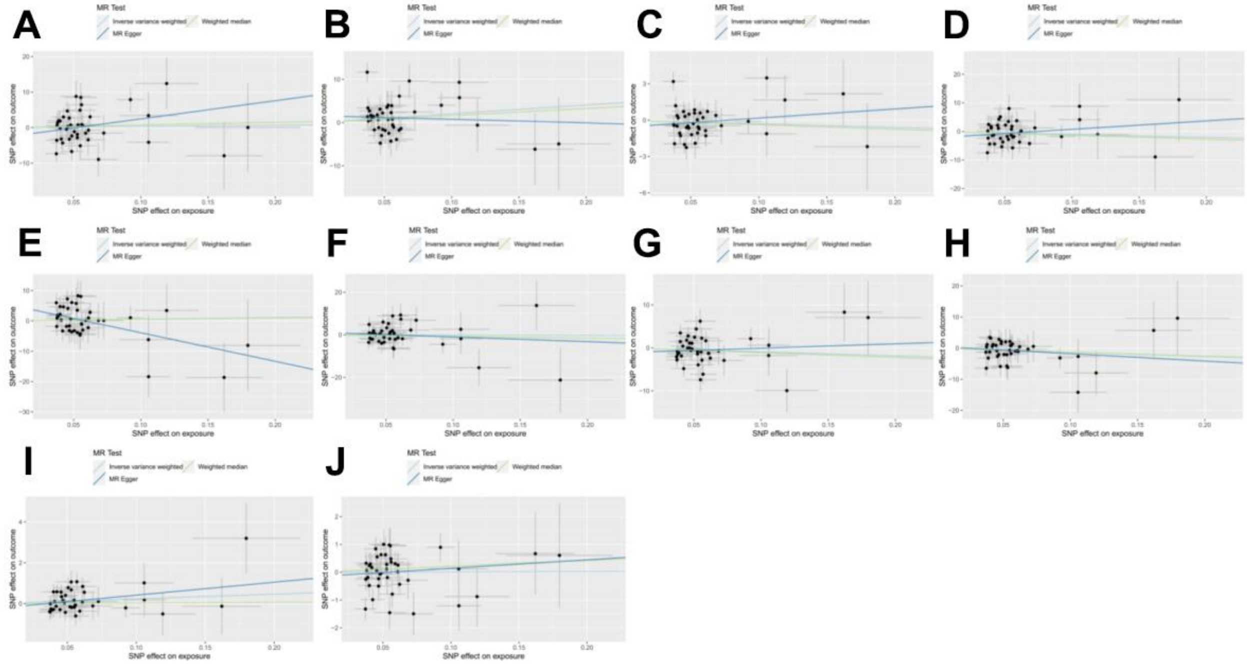


**Supplementary Figure 10. MR scatter plot 3 of the relationship between heart failure and the cortical surface area of functional brain areas.** (**A**) Precentral, (**B**) precuneus, (**C**) rostral anterior cingulate, (**D**) rostral middle frontal, (**E**) superior frontal, (**F**) superior parietal, (**G**) superior temporal, (**H**) supramarginal, (**I**) temporal pole, (**J**) transverse temporal.

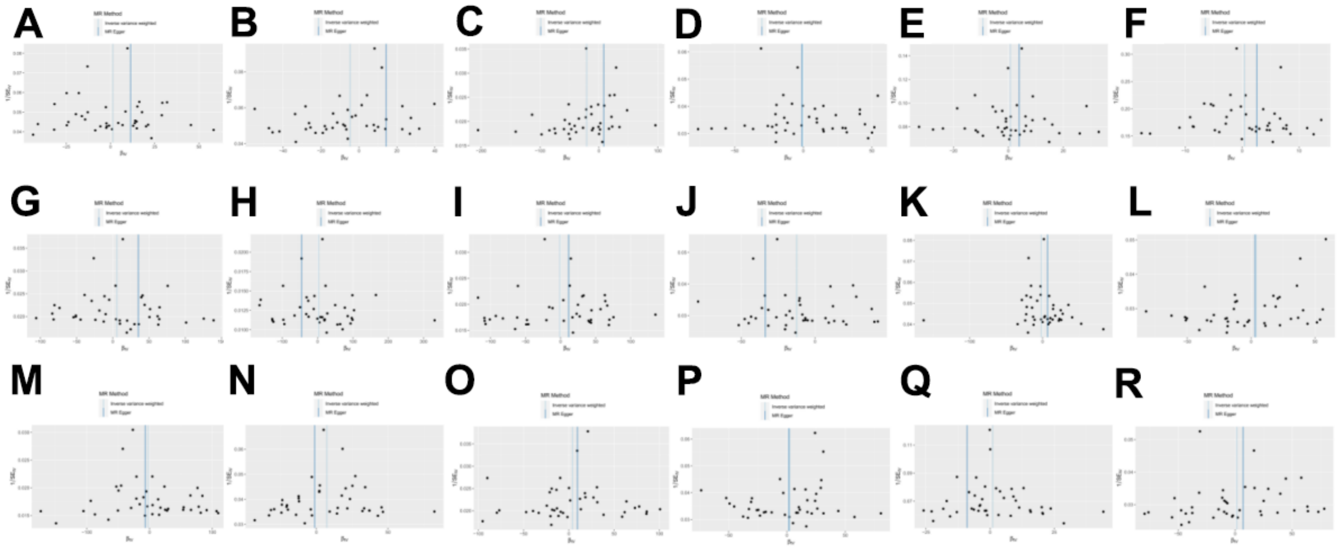


**Supplementary Figure 11. Funnel plot 1 of heterogeneity test results of MR analysis of the relationship between heart failure and the cortical surface area of functional cerebral areas.** (**A**) Bankssts, (**B**) caudal anterior cingulate, (**C**) caudal middle frontal, (**D**) cuneus, (**E**) entorhinal, (**F**) frontal pole, (**G**) fusiform, (**H**) inferior parietal, (**I**) inferior temporal, (**J**) insula, (**K**) isthmus cingulate, (**L**) lateral occipital, (**M**) lateral orbitofrontal, (**N**) lingual, (**O**) medial orbitofrontal, (**P**) middle temporal, (**Q**) paracentral, (**R**) parahippocampal.

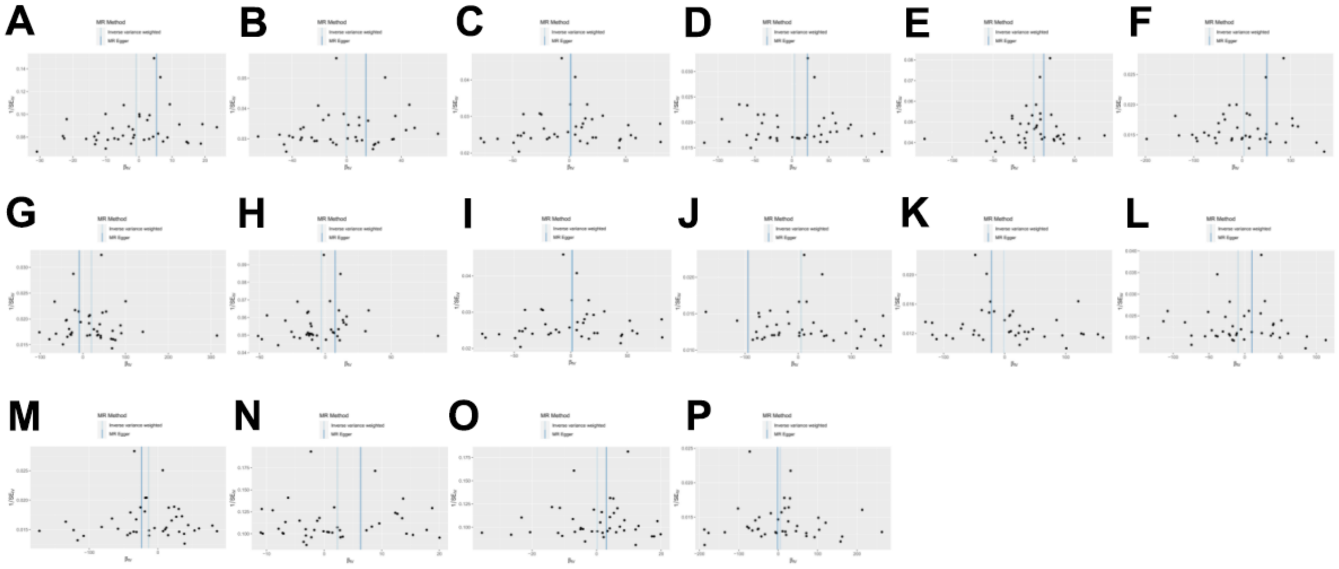


**Supplementary Figure 12. Funnel plot 2 of heterogeneity test results of MR analysis of in the relationship between heart failure and the cortical surface area of functional cerebral areas.** (**A**) Pars opercularis, (**B**) pars orbitalis, (**C**) pars triangularis, (**D**) pericalcarine, (**E**) postcentral, (**F**) posterior cingulate, (**G**) precentral, (**H**) precuneus, (**I**) rostral anterior cingulate, (**J**) rostral middle frontal, (**K**) superior frontal, (**L**) superior parietal, (**M**) superior temporal, (**N**) supramarginal, (**O**) temporal pole, (**P**) transverse temporal.

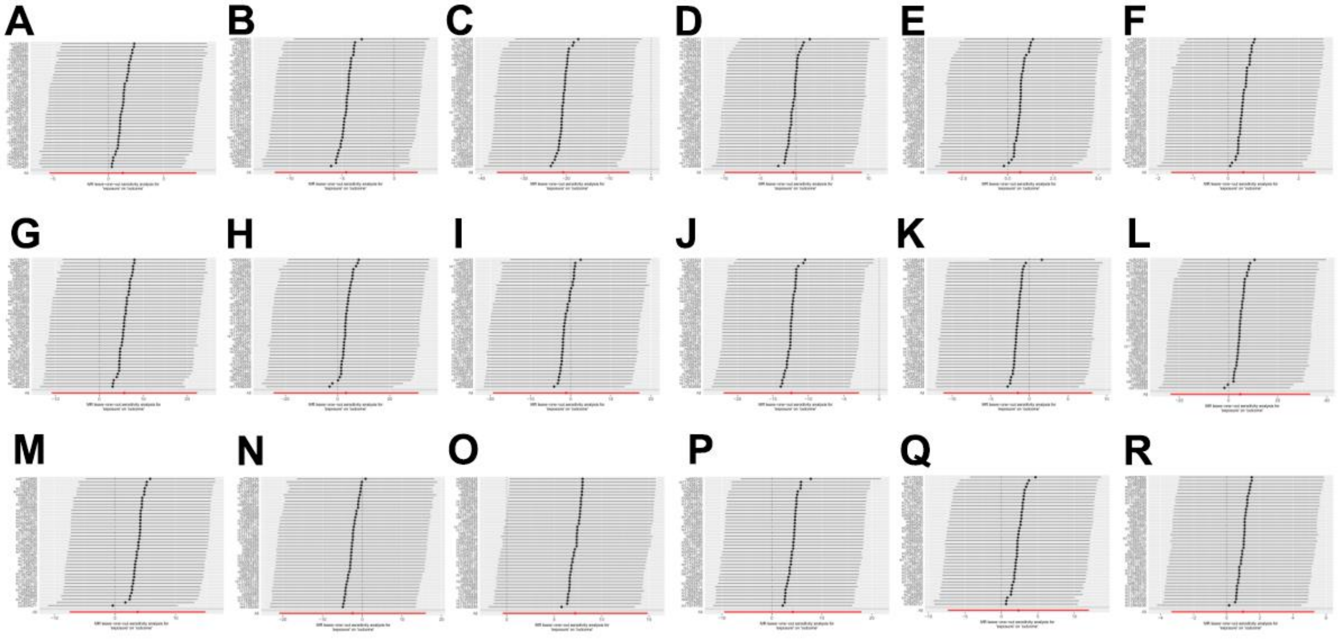

**Supplementary Figure 13. “Leave-one-out” forest map 1 of MR analysis of the relationship between heart failure and the cortical surface area of functional brain areas.** (**A**) Bankssts, (**B**) caudal anterior cingulate, (**C**) caudal middle frontal, (**D**) cuneus, (**E**) entorhinal, (**F**) frontal pole, (**G**) fusiform, (**H**) inferior parietal, (**I**) inferior temporal, (**J**) insula, (**K**) isthmus cingulate, (**L**) lateral occipital, (**M**) lateral orbitofrontal, (**N**) lingual, (**O**) medial orbitofrontal, (**P**) middle temporal, (**Q**) paracentral, (**R**) parahippocampal.

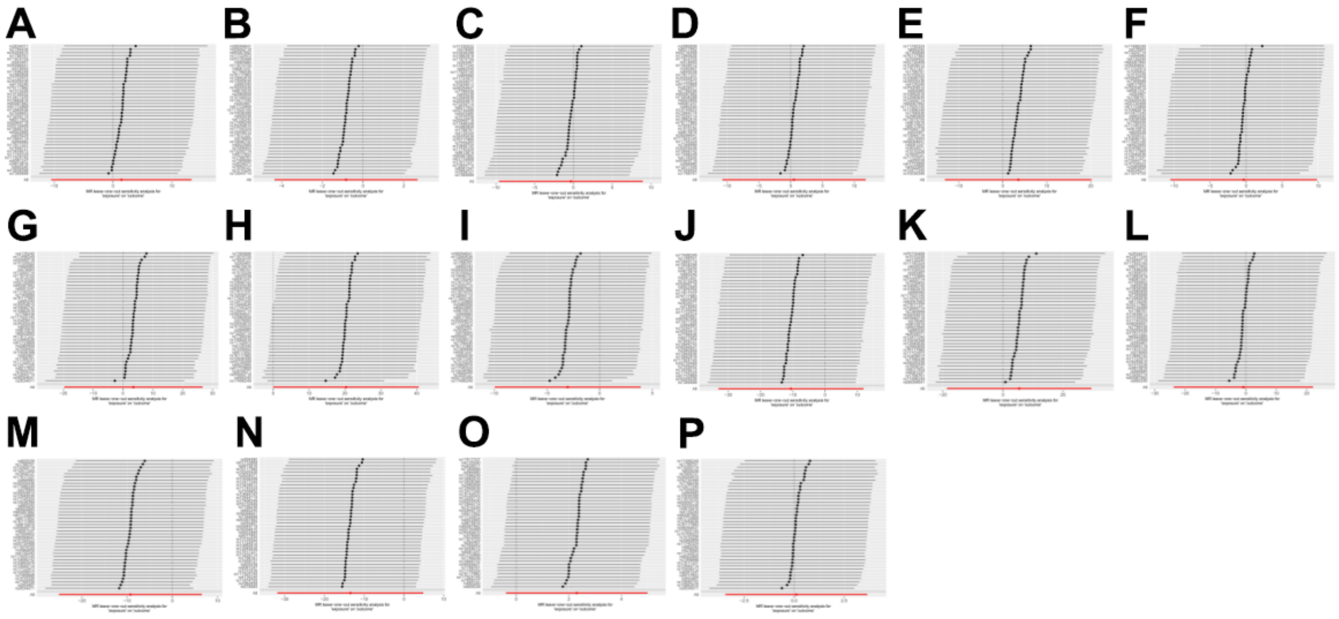

**Supplementary Figure 14. "Leave-one-out" forest map 2 of MR analysis of the relationship between heart failure and the cortical surface of functional brain areas.** (**A**) Pars opercularis, (**B**) pars orbitalis, (**C**) pars triangularis, (**D**) pericalcarine, (**E**) postcentral, (**F**) posterior cingulate, (**G**) precentral, (**H**) precuneus, (**I**) rostral anterior cingulate, (**J**) rostral middle frontal, (**K**) superior frontal, (**L**) superior parietal, (**M**) superior temporal, (**N**) supramarginal, (**O**) temporal pole, (**P**) transverse temporal.
