# Supplementary material for: Heart failure potentially affects the cortical structure of the brain
Source: Aging (Albany NY). 2024 Apr 22;16(8):7357–86. doi: 10.18632/aging.205762 (PMC11087114; doi:10.18632/aging.205762)
Supplement: Supplementary Tables [file aging-16-205762-s002.pdf]

## SUPPLEMENTARY TABLES

**Supplementary Table 1. Basic information of SNPs associated with heart failure.**

| SNPs | other_allele | effect_allele | *β* | *SE* | *P* |
|---|---|---|---|---|---|
| rs55751848 | C | G | -0.0425 | 0.0089 | 1.79E-06 |
| rs593467 | G | A | -0.0548 | 0.0118 | 3.42E-06 |
| rs660240 | T | C | 0.0611 | 0.0097 | 3.00E-10 |
| rs35054810 | G | A | 0.0725 | 0.0143 | 3.98E-07 |
| rs7559452 | A | G | 0.0468 | 0.0102 | 4.47E-06 |
| rs17496249 | A | G | -0.0372 | 0.0079 | 2.49E-06 |
| rs12477245 | C | T | 0.1192 | 0.0236 | 4.40E-07 |
| rs7369998 | G | A | -0.059 | 0.0126 | 2.83E-06 |
| rs72844714 | C | A | 0.0559 | 0.0121 | 3.84E-06 |
| rs80087882 | G | A | 0.0609 | 0.0125 | 1.10E-06 |
| rs4376020 | T | A | -0.0612 | 0.0123 | 6.50E-07 |
| rs9815816 | T | C | 0.0479 | 0.0099 | 1.31E-06 |
| rs10938398 | G | A | 0.0389 | 0.008 | 1.16E-06 |
| rs11722972 | T | G | -0.0519 | 0.0114 | 5.30E-06 |
| rs2634071 | T | C | -0.0923 | 0.0101 | 6.33E-20 |
| rs11745324 | G | A | -0.0528 | 0.0095 | 2.73E-08 |
| rs7766436 | C | T | 0.04 | 0.0086 | 3.30E-06 |
| rs4135240 | T | C | -0.0486 | 0.0084 | 7.22E-09 |
| rs6922885 | T | C | -0.0377 | 0.008 | 2.45E-06 |
| rs55949718 | C | T | -0.0685 | 0.0142 | 1.41E-06 |
| rs1510226 | T | C | 0.162 | 0.0285 | 1.31E-08 |
| rs55730499 | C | T | 0.1058 | 0.0157 | 1.60E-11 |
| rs117925145 | A | G | 0.1797 | 0.0391 | 4.31E-06 |
| rs76117960 | T | C | 0.0528 | 0.0113 | 2.97E-06 |
| rs35005436 | T | C | 0.0533 | 0.0116 | 4.33E-06 |
| rs10952517 | T | A | -0.0394 | 0.0085 | 3.56E-06 |
| rs73200714 | G | A | -0.055 | 0.0118 | 3.15E-06 |
| rs2980858 | T | C | -0.04 | 0.0086 | 3.30E-06 |
| rs7859727 | C | T | 0.0623 | 0.0078 | 1.38E-15 |
| rs600038 | T | C | 0.0569 | 0.0096 | 3.08E-09 |
| rs994980 | C | T | 0.0375 | 0.0081 | 3.66E-06 |
| rs72807031 | T | A | -0.0895 | 0.0181 | 7.62E-07 |
| rs4746140 | G | C | -0.0666 | 0.0109 | 9.96E-10 |
| rs10882816 | G | T | -0.0447 | 0.0085 | 1.45E-07 |
| rs17617337 | C | T | -0.0561 | 0.0095 | 3.52E-09 |
| rs61733868 | T | C | -0.1057 | 0.0216 | 9.90E-07 |
| rs186973337 | T | A | 0.0933 | 0.0196 | 1.93E-06 |
| rs4755717 | C | G | -0.0379 | 0.008 | 2.16E-06 |
| rs10459012 | C | A | 0.0458 | 0.0095 | 1.43E-06 |
| rs12805761 | A | T | -0.0672 | 0.0139 | 1.33E-06 |
| rs4766578 | T | A | -0.0433 | 0.0079 | 4.23E-08 |
| rs10846742 | G | A | -0.0506 | 0.0111 | 5.15E-06 |
| rs8017852 | C | A | -0.0554 | 0.012 | 3.90E-06 |
| rs10150022 | A | G | -0.0419 | 0.0087 | 1.46E-06 |

| | | | | | |
|---|---|---|---|---|---|
| rs17483686 | A | T | 0.0489 | 0.0095 | 2.64E-07 |
| rs56094641 | A | G | 0.0454 | 0.008 | 1.39E-08 |
| rs578065 | T | G | 0.0408 | 0.0082 | 6.50E-07 |
| rs12940636 | T | C | -0.0381 | 0.0083 | 4.42E-06 |
| rs8081247 | G | A | -0.0487 | 0.0098 | 6.72E-07 |
| rs1788761 | A | G | -0.0425 | 0.009 | 2.33E-06 |
| rs11874705 | A | G | 0.0469 | 0.0098 | 1.70E-06 |
| rs10520390 | C | G | -0.0902 | 0.0187 | 1.41E-06 |

**Supplementary Table 2. MR results of the effect of heart failure on the cortical structure of functional cerebral areas.**

| | SA | TH |
|---|---|---|
| | Global weighted | |
| Bankssts | 0.6977748 | 0.9969515 |
| Caudal anterior cingulate | 0.1879553 | 0.9944418 |
| Caudal middle frontal | 0.009369889 | 0.4861102 |
| Cuneus | 0.93282 | 0.3884087 |
| Entorhinal | 0.7375308 | 0.4824811 |
| Frontal pole | 0.6806647 | 0.9700872 |
| Fusiform | 0.5006216 | 0.8486323 |
| Inferior parietal | 0.8200912 | 0.2974922 |
| Inferior temporal | 0.9055626 | 0.1172891 |
| Insula | 0.01088126 | 0.2536509 |
| Isthmus cingulate | 0.7616352 | 0.8956049 |
| Lateral occipital | 0.740052 | 0.6561721 |
| Lateral orbitofrontal | 0.5156932 | 0.1242299 |
| Lingual | 0.7964685 | 0.3125903 |
| Medial orbitofrontal | 0.06407264 | 0.6340118 |
| Middle temporal | 0.5498127 | 0.6994723 |
| Paracentral | 0.6369187 | 0.6598778 |
| Parahippocampal | 0.6559629 | 0.7828665 |
| Pars opercularis | 0.8144172 | 0.6971543 |
| Par orbitalis | 0.6418063 | 0.4258328 |
| Pars triangularis | 0.9436559 | 0.5506617 |
| Pericalcarine | 0.9299025 | 0.4058616 |
| Postcentral | 0.6802846 | 0.8207215 |
| Posterior cingulate | 0.9478465 | 0.3376118 |
| Precentral | 0.7710834 | 0.773043 |
| Precuneus | 0.0496107 | 0.3306158 |
| Rostral anterior cingulate | 0.3899105 | 0.6066478 |
| Rostral middle frontal | 0.3578567 | 0.2814057 |
| Superior frontal | 0.66020664 | 0.5205543 |
| Superior parietal | 0.9424974 | 0.04477682 |
| Superior temporal | 0.2498117 | 0.843193 |
| Supramarginal | 0.1505051 | 0.4930569 |
| Temporal pole | 0.09355074 | 0.6033848 |
| Transverse temporal | 0.9531146 | 0.1976769 |

**Supplementary Table 3. MR pleiotropic analysis of cortical thickness in cerebral functional areas in heart failure patients.**

| Cerebral cortex | Egger-intercept | *SE* | *P* |
|---|---|---|---|
| Bankssts | -0.000151779 | 0.000882834 | 0.8643876 |
| Caudal anterior cingulate | 0.000487773 | 0.00104738 | 0.6440152 |
| Caudal middle frontal | -0.000299749 | 0.000605053 | 0.623091 |
| Cuneus | -0.000922428 | 0.000574205 | 0.1162442 |
| Entorhinal | -0.002141749 | 0.001745485 | 0.22717 |
| Frontal pole | 0.000195246 | 0.001289201 | 0.8804034 |
| Fusiform | -0.000658134 | 0.000539236 | 0.2296064 |
| Inferior parietal | 0.000226421 | 0.0004656 | 0.6294766 |
| Inferior temporal | -0.0002893 | 0.000607283 | 0.6364592 |
| Insula | -0.000799705 | 0.000735893 | 0.2838346 |
| Isthmus cingulate | 0.000161048 | 0.000943923 | 0.865408 |
| Lateral occipital | 0.000234886 | 0.000612812 | 0.7035848 |
| Lateral orbitofrontal | -0.000424334 | 0.000596338 | 0.4809688 |
| Lingual | 0.000286671 | 0.000493235 | 0.5644465 |
| Medial orbitofrontal | -0.000220342 | 0.000722794 | 0.7621033 |
| Middle temporal | 0.000546603 | 0.000566318 | 0.3403976 |
| Paracentral | -0.000464085 | 0.000608095 | 0.4499489 |
| Parahippocampal | -0.000661862 | 0.001500857 | 0.6616559 |
| Pars opercularis | -0.00073961 | 0.000494173 | 0.1425313 |
| Pars orbitalis | -0.000512167 | 0.0008384 | 0.5448189 |
| Pars triangularis | 0.000264559 | 0.000619918 | 0.671898 |
| Pericalcarine | 0.000108325 | 0.000558845 | 0.847309 |
| Postcentral | -6.45318E-05 | 0.000631108 | 0.9190809 |
| Posterior cingulate | 0.000420647 | 0.000618175 | 0.5002296 |
| Precentral | 6.53192E-05 | 0.000544684 | 0.9051611 |
| Precuneus | -0.000272092 | 0.000417158 | 0.5180639 |
| Rostral anterior cingulate | -0.000270701 | 0.000929568 | 0.7724327 |
| Rostral middle frontal | 0.000424245 | 0.000443086 | 0.3442245 |
| Superior frontal | -0.000272435 | 0.000581861 | 0.6422378 |
| Superior parietal | 2.09165E-05 | 0.000411503 | 0.9597207 |
| Superior temporal | -0.000939761 | 0.000581243 | 0.1139801 |
| Supramarginal | 6.47205E-06 | 0.00040376 | 0.9872926 |
| Temporal pole | -0.000355457 | 0.001417925 | 0.803371 |
| Transverse temporal | -0.000251782 | 0.000907365 | 0.7828721 |

**Supplementary Table 4. MR pleiotropic analysis of cortical surface area of cerebral functional area in heart failure.**

| Cerebral cortex | Egger-intercept | *SE* | *P* |
|---|---|---|---|
| Bankssts | -0.5550066 | 0.6080988 | 0.3670126 |
| Caudal anterior cingulate | -1.047564 | 0.6136488 | 0.09575322 |
| Caudal middle frontal | -1.608463 | 1.42652 | 0.2664022 |
| Cuneus | 0.03487043 | 0.8842244 | 0.9687438 |
| Entorhinal | -0.1806707 | 0.3692631 | 0.6273886 |
| Frontal pole | -0.1204245 | 0.189236 | 0.5282514 |
| Fusiform | -1.631361 | 1.515063 | 0.2882028 |
| Inferior parietal | 2.67364 | 2.543369 | 0.2996279 |
| Inferior temporal | -0.6900998 | 1.686612 | 0.684658 |
| Insula | 1.182193 | 0.8740075 | 0.1839702 |
| Isthmus cingulate | -0.4546133 | 0.8927734 | 0.613471 |
| Lateral occipital | 0.3568113 | 2.634551 | 0.8929643 |
| Lateral orbitofrontal | 0.03948283 | 1.03887 | 0.9698771 |
| Lingual | 0.2514821 | 1.712191 | 0.8839855 |
| Medial orbitofrontal | 0.4619694 | 0.703708 | 0.5153712 |
| Middle temporal | -0.2951111 | 1.265819 | 0.8168732 |
| Paracentral | 0.04591706 | 0.8976835 | 0.9594666 |
| Parahippocampal | 0.5518967 | 0.394956 | 0.1702041 |
| Pars opercularis | -0.3101397 | 1.105776 | 0.7805976 |
| Pars orbitalis | -0.3421035 | 0.321653 | 0.2940605 |
| Pars triangularis | -0.7926667 | 0.8503531 | 0.356988 |
| Pericalcarine | -0.04470478 | 1.03377 | 0.9657274 |
| Postcentral | -0.9813764 | 1.542445 | 0.5283318 |
| Posterior cingulate | -0.68538 | 0.9402103 | 0.4703785 |
| Precentral | -2.642603 | 2.116006 | 0.2191595 |
| Precuneus | 1.577018 | 1.865732 | 0.4031248 |
| Rostral anterior cingulate | -0.5860225 | 0.6358936 | 0.3624173 |
| Rostral middle frontal | -2.219558 | 2.060125 | 0.2879254 |
| Superior frontal | 5.515524 | 2.069316 | 0.01112991 |
| Superior parietal | 1.161043 | 2.1021 | 0.5838773 |
| Superior temporal | -1.075803 | 1.45459 | 0.4639763 |
| Supramarginal | 0.5605873 | 1.683823 | 0.7409738 |
| Temporal pole | -0.2244677 | 0.2469013 | 0.3688609 |
| Transverse temporal | -0.1623271 | 0.3284371 | 0.6239085 |

**Supplementary Table 5. MR sensitivity analysis of cortical thickness in cerebral functional areas in heart failure patients.**

| Cerebral cortex | Method | Cochran's Q | *P* |
|---|---|---|---|
| Bankssts | MR–Egger | 75.7908 | 0.00037696 |
| | Inverse variance weighted | 75.84828 | 0.000533208 |
| Caudal anterior cingulate | MR–Egger | 33.69524 | 0.7101431 |
| | Inverse variance weighted | 33.91213 | 0.7398873 |
| Caudal middle frontal | MR–Egger | 56.01457 | 0.03798923 |
| | Inverse variance weighted | 56.36708 | 0.04463329 |
| Cuneus | MR–Egger | 35.3256 | 0.6382164 |
| | Inverse variance weighted | 37.90626 | 0.5648783 |
| Entorhinal | MR–Egger | 45.01153 | 0.2347797 |
| | Inverse variance weighted | 46.74919 | 0.2149053 |
| Frontal pole | MR–Egger | 51.17802 | 0.09164378 |
| | Inverse variance weighted | 51.20812 | 0.11026081 |
| Fusiform | MR–Egger | 33.8945 | 0.7015977 |
| | Inverse variance weighted | 35.3841 | 0.6779649 |
| Inferior parietal | MR–Egger | 54.50738 | 0.05060953 |
| | Inverse variance weighted | 54.8379 | 0.05916865 |
| Inferior temporal | MR–Egger | 37.05187 | 0.559015 |
| | Inverse variance weighted | 37.27881 | 0.593428 |
| Insula | MR–Egger | 54.74393 | 0.04841658 |
| | Inverse variance weighted | 56.40161 | 0.04434429 |
| Isthmus cingulate | MR–Egger | 43.8885 | 0.272001 |
| | Inverse variance weighted | 43.92134 | 0.3089267 |
| Lateral occipital | MR–Egger | 66.80433 | 0.003656039 |
| | Inverse variance weighted | 67.05598 | 0.004675132 |
| Lateral orbitofrontal | MR–Egger | 37.63968 | 0.5319041 |
| | Inverse variance weighted | 38.14601 | 0.5539554 |
| Lingual | MR–Egger | 25.74057 | 0.9492973 |
| | Inverse variance weighted | 26.07837 | 0.9562573 |
| Medial orbitofrontal | MR–Egger | 43.73081 | 0.2775087 |
| | Inverse variance weighted | 43.83502 | 0.3121266 |
| Middle temporal | MR–Egger | 35.21808 | 0.643078 |
| | Inverse variance weighted | 36.14967 | 0.644267 |
| Paracentral | MR–Egger | 43.53853 | 0.2843121 |
| | Inverse variance weighted | 44.18875 | 0.2991325 |
| Parahippocampal | MR–Egger | 44.23944 | 0.2599963 |
| | Inverse variance weighted | 44.46003 | 0.2893824 |
| Pars opercularis | MR–Egger | 40.94209 | 0.3852885 |
| | Inverse variance weighted | 43.29363 | 0.3326096 |
| Pars orbitalis | MR–Egger | 46.3846 | 0.1940741 |
| | Inverse variance weighted | 46.82844 | 0.2125827 |
| Pars triangularis | MR–Egger | 54.73514 | 0.04849656 |
| | Inverse variance weighted | 54.99075 | 0.05755383 |
| Pericalcarine | MR–Egger | 40.9379 | 0.3854638 |

| | | | |
|---|---|---|---|
| | Inverse variance weighted | 40.97734 | 0.4274514 |
| Postcentral | MR–Egger | 72.35742 | 0.000925439 |
| | Inverse variance weighted | 72.37682 | 0.001294442 |
| Posterior cingulate | MR–Egger | 39.18783 | 0.4614509 |
| | Inverse variance weighted | 39.65309 | 0.4857295 |
| Precentral | MR–Egger | 47.12074 | 0.1744296 |
| | Inverse variance weighted | 47.13811 | 0.2036735 |
| Precuneus | MR–Egger | 39.60568 | 0.4428657 |
| | Inverse variance weighted | 40.03772 | 0.4685824 |
| Rostral anterior cingulate | MR–Egger | 33.30739 | 0.7265343 |
| | Inverse variance weighted | 33.39219 | 0.760587 |
| Rostral middle frontal | MR–Egger | 31.84448 | 0.7849585 |
| | Inverse variance weighted | 32.76124 | 0.7847206 |
| Superior frontal | MR–Egger | 62.21916 | 0.01047296 |
| | Inverse variance weighted | 62.5689 | 0.0127607 |
| Superior parietal | MR–Egger | 38.93501 | 0.4728037 |
| | Inverse variance weighted | 38.9376 | 0.5179703 |
| Superior temporal | MR–Egger | 41.86421 | 0.3475959 |
| | Inverse variance weighted | 44.67028 | 0.2819567 |
| Supramarginal | MR–Egger | 33.2165 | 0.7303263 |
| | Inverse variance weighted | 33.21675 | 0.7674103 |
| Temporal pole | MR–Egger | 39.35957 | 0.4537835 |
| | Inverse variance weighted | 39.423 | 0.4960549 |
| Transverse temporal | MR–Egger | 29.46499 | 0.86554 |
| | Inverse variance weighted | 29.54199 | 0.8875942 |

**Supplementary Table 6. MR sensitivity analysis of cortical surface area of cerebral functional area in heart failure patients.**

| Cerebral cortex | Method | Cochran's Q | *P* |
|---|---|---|---|
| Bankssts | MR–Egger | 43.45859 | 0.2871696 |
| | Inverse variance weighted | 44.38683 | 0.2919947 |
| Caudal anterior cingulate | MR–Egger | 55.62494 | 0.04095567 |
| | Inverse variance weighted | 59.78143 | 0.02287035 |
| Caudal middle frontal | MR–Egger | 43.39232 | 0.2895516 |
| | Inverse variance weighted | 44.80686 | 0.2771945 |
| Cuneus | MR–Egger | 50.20767 | 0.1077826 |
| | Inverse variance weighted | 50.20967 | 0.1292751 |
| Entorhinal | MR–Egger | 50.44128 | 0.1037027 |
| | Inverse variance weighted | 50.75089 | 0.1186733 |
| Frontal pole | MR–Egger | 59.94121 | 0.01713385 |
| | Inverse variance weighted | 60.56363 | 0.01948027 |
| Fusiform | MR–Egger | 53.95883 | 0.05602575 |
| | Inverse variance weighted | 55.56295 | 0.05183819 |
| Inferior parietal | MR–Egger | 52.14915 | 0.07752271 |
| | Inverse variance weighted | 53.62679 | 0.07337278 |

| | | | |
|---|---|---|---|
| Inferior temporal | MR–Egger | 52.0202 | 0.07928585 |
| | Inverse variance weighted | 52.24359 | 0.09296391 |
| Insula | MR–Egger | 28.86444 | 0.8826167 |
| | Inverse variance weighted | 30.694 | 0.8549859 |
| Isthmus cingulate | MR–Egger | 89.33176 | 8.04E-06 |
| | Inverse variance weighted | 89.92571 | 1.05E-05 |
| Lateral occipital | MR–Egger | 71.9915 | 0.00101626 |
| | Inverse variance weighted | 72.02536 | 0.001413118 |
| Lateral orbitofrontal | MR–Egger | 47.4027 | 0.1673007 |
| | Inverse variance weighted | 47.40452 | 0.1962198 |
| Lingual | MR–Egger | 46.89388 | 0.1803229 |
| | Inverse variance weighted | 46.91982 | 0.2099263 |
| Medial orbitofrontal | MR–Egger | 39.32461 | 0.4553414 |
| | Inverse variance weighted | 39.75916 | 0.4809858 |
| Middle temporal | MR–Egger | 39.46136 | 0.4492581 |
| | Inverse variance weighted | 39.51635 | 0.4918601 |
| Paracentral | MR–Egger | 53.83827 | 0.05728037 |
| | Inverse variance weighted | 53.84188 | 0.07065973 |
| Parahippocampal | MR–Egger | 34.00666 | 0.6967528 |
| | Inverse variance weighted | 35.95928 | 0.6527204 |
| Pars opercularis | MR–Egger | 58.4168 | 0.02353054 |
| | Inverse variance weighted | 58.53463 | 0.02937326 |
| Pars orbitalis | MR–Egger | 39.98337 | 0.4262844 |
| | Inverse variance weighted | 41.14309 | 0.4203288 |
| Pars triangularis | MR–Egger | 40.17411 | 0.417997 |
| | Inverse variance weighted | 41.0692 | 0.4234989 |
| Pericalcarine | MR–Egger | 38.9605 | 0.4716557 |
| | Inverse variance weighted | 38.96237 | 0.516848 |
| Postcentral | MR–Egger | 43.23858 | 0.2951222 |
| | Inverse variance weighted | 43.68739 | 0.3176415 |
| Posterior cingulate | MR–Egger | 98.71798 | 4.41E-07 |
| | Inverse variance weighted | 100.06305 | 4.70E-07 |
| Precentral | MR–Egger | 58.99376 | 0.0208929 |
| | Inverse variance weighted | 61.353 | 0.01652484 |
| Precuneus | MR–Egger | 62.79128 | 0.009224499 |
| | Inverse variance weighted | 63.94157 | 0.009463078 |
| Rostral anterior cingulate | MR–Egger | 63.72689 | 0.00747466 |
| | Inverse variance weighted | 65.11466 | 0.007286857 |
| Rostral middle frontal | MR–Egger | 30.68133 | 0.8268206 |
| | Inverse variance weighted | 31.8421 | 0.8177259 |
| Superior frontal | MR–Egger | 38.32725 | 0.5003717 |
| | Inverse variance weighted | 45.43153 | 0.2560455 |
| Superior parietal | MR–Egger | 36.7003 | 0.5752397 |
| | Inverse variance weighted | 37.00544 | 0.6058215 |
| Superior temporal | MR–Egger | 55.883 | 0.03896919 |
| | Inverse variance weighted | 56.66679 | 0.04217886 |

| | | | |
|---|---|---|---|
| Supramarginal | MR–Egger | 31.81319 | 0.786142 |
| | Inverse variance weighted | 31.92403 | 0.8148943 |
| Temporal pole | MR–Egger | 39.5041 | 0.4473617 |
| | Inverse variance weighted | 40.34132 | 0.4551645 |
| Transverse temporal | MR–Egger | 61.30767 | 0.01278561 |
| | Inverse variance weighted | 61.69167 | 0.01538625 |
